# Tuning Active Metal Atomic Spacing by Filling of Light Atoms and Resulting Reversed Hydrogen Adsorption-Distance Relationship for Efficient Catalysis

**DOI:** 10.1007/s40820-023-01142-1

**Published:** 2023-07-03

**Authors:** Ding Chen, Ruihu Lu, Ruohan Yu, Hongyu Zhao, Dulan Wu, Youtao Yao, Kesong Yu, Jiawei Zhu, Pengxia Ji, Zonghua Pu, Zongkui Kou, Jun Yu, Jinsong Wu, Shichun Mu

**Affiliations:** 1 State Key Laboratory of Advanced Technology for Materials Synthesis and Processing, Wuhan University of Technology, Wuhan, 430070 People’s Republic of China; 2 NRC (Nanostructure Research Centre), Wuhan University of Technology, Wuhan, 430070 People’s Republic of China

**Keywords:** Electrocatalysis, DFT calculation, Interstitial filling, Hydrogen evolution, Structure–activity relationships

## Abstract

Precisely tuning the spacing of the active centers on the atomic scale is of great significance to improve the catalytic activity and deepen the understanding of the catalytic mechanism, but still remains a challenge. Here, we develop a strategy to dilute catalytically active metal interatomic spacing (d_M-M_) with light atoms and discover the unusual adsorption patterns. For example, by elevating the content of boron as interstitial atoms, the atomic spacing of osmium (d_Os-Os_) gradually increases from 2.73 to 2.96 Å. More importantly, we find that, with the increase in d_Os-Os_, the hydrogen adsorption-distance relationship is reversed via downshifting *d*-band states, which breaks the traditional cognition, thereby optimizing the H adsorption and H_2_O dissociation on the electrode surface during the catalytic process; this finally leads to a nearly linear increase in hydrogen evolution reaction activity. Namely, the maximum d_Os-Os_ of 2.96 Å presents the optimal HER activity (8 mV @ 10 mA cm^−2^) in alkaline media as well as suppressed O adsorption and thus promoted stability. It is believed that this novel atomic-level distance modulation strategy of catalytic sites and the reversed hydrogen adsorption-distance relationship can shew new insights for optimal design of highly efficient catalysts.

**Supplementary Information:**

The online version contains supplementary material available at 10.1007/s40820-023-01142-1.

## Highlights

- Density functional theory calculations indicates that interstitial B atoms can tune the atomic spacing of host metal Os and achieve a reversal hydrogen adsorption-distance relationship.
- The structure–activity relationship between the spacing of active Os atoms and catalytic activity is established.
- Prepared OsB_2_ with increasing d_Os-Os_ of 2.96 Å presents the optimal hydrogen evolution reaction activity (8 mV @ 10 mA cm^−2^) and robust stability in alkaline media.

**Supplementary Information:**

The online version contains supplementary material available at 10.1007/s40820-023-01142-1.

## Graphical abstract

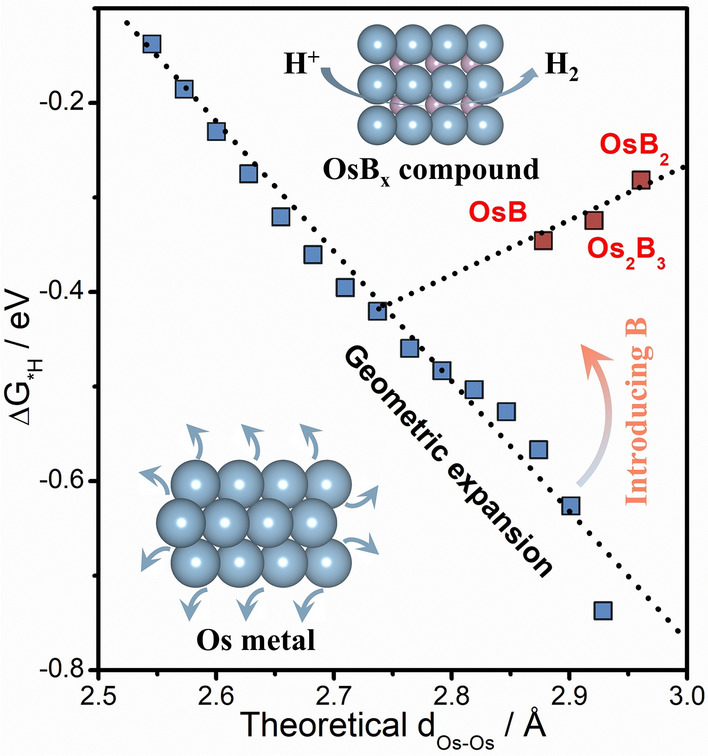

## Introduction

Hitherto, it has been found that when the spacing between active centers is minimized to the atomic scale, their interaction would have a strong impact on the catalytic process [1–6]. For instance, atomically dispersed Fe^3+^ sites can accelerate electroreduction of CO_2_ to CO [7], the inter-site distance effect of isolated Fe-N_4_ is the origin of the enhancement of the oxygen reduction activity [8], and the regulation of mean distance between Rh atoms has a synergistic catalytic effect on hydrogen evolution reaction (HER) [9]. However, current research focuses on the modulation of site spacing by controlling the active site density or applying stress, which is difficult for precisely tailoring, so as hindering comprehensive understanding of the site distance dominated reaction pathways. Furthermore, unveiling the reaction mechanism at the atomic scale is the fundamental way to improve the catalytic efficiency [10–14]. Therefore, to establish the structure–activity relationship of target catalytic reactions, an advanced and unique atom spacing modulation strategy is very worthy of in-depth investigation.

Theoretically, light atoms (H, B, C) occupying interstitial sites in the metal matrix lattice could tune the spacing and electronic structure of host atoms well [15–18]. Now, some works have shown that the interstitial light atoms can cause a certain lattice expansion, improving the adsorption and activity for catalysts [19, 20]. Unfortunately, it is difficult to control the content and order of interstitial light elements experimentally [21]. In addition, the penetration of light elements into the tightly packed metal lattice requires a highly activated process, leading to the extremely high synthesis condition and the sintering of metal nanoparticles [22]. Consequently, using an interval-filling strategy to achieve controllable gradient dispersion of active atoms, with a low energy barrier, could be considered, but it has always been a research blank.

Herein, in terms of density functional theory (DFT) calculations, we first confirm that B atoms with small radius and low electronegativity can perfectly balance the stress change and electron transfer during geometric expansion of metallic osmium (Os) as the cheapest Pt-group metal hitherto but excessive adsorbability in electrocatalysis (Table S1); this results in a gradual increase in atomic spacing of Os (d_Os-Os_) from 2.73 to 2.96 Å accompanying increasing amount of B as interstitial atoms in intermetallic OsB_*x*_ (*x* = 1, 1.5, 2) and a reversed hydrogen adsorption-distance relationship. Then, intermetallic B insertion into the Os metal lattice is experimentally realized and orderly gradient arrangement is achieved, forming stable Os-B intermetallic compounds. Unlike conventional surface modification and doping, the strong host–guest electron interactions and the formation of new chemical bonds here further co-enhances the active and stability of the catalyst. Finally, combining in-depth theoretical analysis with detailed experimental characterizations, the structure–activity relationship is established: the spacing of active Os atoms increases with the gradual filling of B, leading to decrease in H binding and H_2_O dissociation barriers. Meanwhile, the enhanced Os-B coordination effect inhibits the deactivation and dissolution of Os, achieving the most active and stable HER catalyst to date.

## Experimental Section

### Material Syntheses

OsB_*x*_ was obtained via one-pot molten salt-assisted route. First, KCl-LiCl (*n*_KCl_:*n*_LiCl_ = 4.1:5.9) were evenly mixed in a glovebox to form the eutectic salt system. Then, 1 mmol Os powder and excessive NaBH_4_ were added in 2.5 g eutectic salts and the mixture was grinded uniformly under the hydrophobic and anaerobic atmosphere. Then mixture was added into a corundum boat and heated for 4 h at T °C under inert atmosphere. After cooled to room temperature, the reaction product was collected and washed with DI water to remove the residual eutectic salts. Finally, after vacuum drying, different phases and crystal forms of osmium boride, namely OsB_*x*_, were obtained. The formation of different phases here depends on the temperature. When T is 700, 800, and 900 °C, the obtained phases are hexagonal phase OsB (OsB-H), hexagonal phase Os_2_B_3_ (Os_2_B_3_-H), and orthorhombic phase OsB_2_ (OsB_2_-O), respectively.

### Material Characterization

X-ray diffraction (XRD) patterns were collected on a Rigaku X-ray diffractometer equipped with a Cu *Kα* radiation source to obtain the crystalline structure of all samples. Inductively coupled plasma (ICP) was used to detect the element content in materials. X-ray photoelectron spectroscopy (XPS), ultraviolet photoelectron spectroscopy (UPS) and X-ray absorption fine structure (EXAFS) were carried out to reveal the electronic structure and valence bond structure. The morphology and structure were characterized by double spherical aberration-corrected scanning transmission electron microscope (AC-STEM, Titan Cubed Themis G2 300).

### Electrochemical Measurements

All electrochemical measurements were performed in a conventional three-electrode system at room temperature using a CHI 660E electrochemical analyzer (CHI Instruments, Shanghai, China). The alkaline (1.0 M KOH) electrochemical measurements were performed using an Ag/AgCl as the reference electrode. The acidic (0.5 M H_2_SO_4_) electrochemical measurements were performed using a saturated calomel electrode (SCE) as the reference electrode. A graphite plate was used as the counter electrode in all measurements. The catalyst ink was prepared by dispersing 4 mg as-prepared sample and 1 mg conductive XC-72 powder into a mixture (900 μL isopropyl alcohol, 100 μL water and 20 μL 5% Nafion solution) and ultrasonic dispersion for 30 min. For comparison, 5 mg commercial catalyst powder (20 wt% Pt/C) was evenly dispersed into the same mixture. Polarization data were obtained at a scan rate of 5 mV s^−1^. All polarization curves were iR-corrected. Electrochemical impedance spectroscopy (EIS) was conducted at the corresponding potentials of 10 mA cm^−2^ from LSV curves, with the frequency range of 0.01 Hz to 100 kHz with AC amplitude of 10 mV. Electrochemical double-layer capacitance (*C*_dl_) was determined with typical CV measurements at various scan rates (20, 40, 60, 80 and 100 mV s^−1^) in nonreactive region. The *C*_dl_ was further employed to obtain the ECSA value according to the equation: ECSA = *C*_dl_/*C*_s_. Turnover frequency (TOF) value for HER was calculated from the following equation: TOF = *jA*/2*nF*, where *j* is the current density estimated from the LSV, *A* stands for the exposed area of applied electrode, *F* is the Faraday constant and *n* is the number of moles of metal content in the electrode. The durability was evaluated by comparing LSV curves before and after CV cycling test and chronoamperometry at the overpotential of 50 mA cm^−2^.

## Results and Discussion

### Theoretical Predictions

**Fig. 1:**
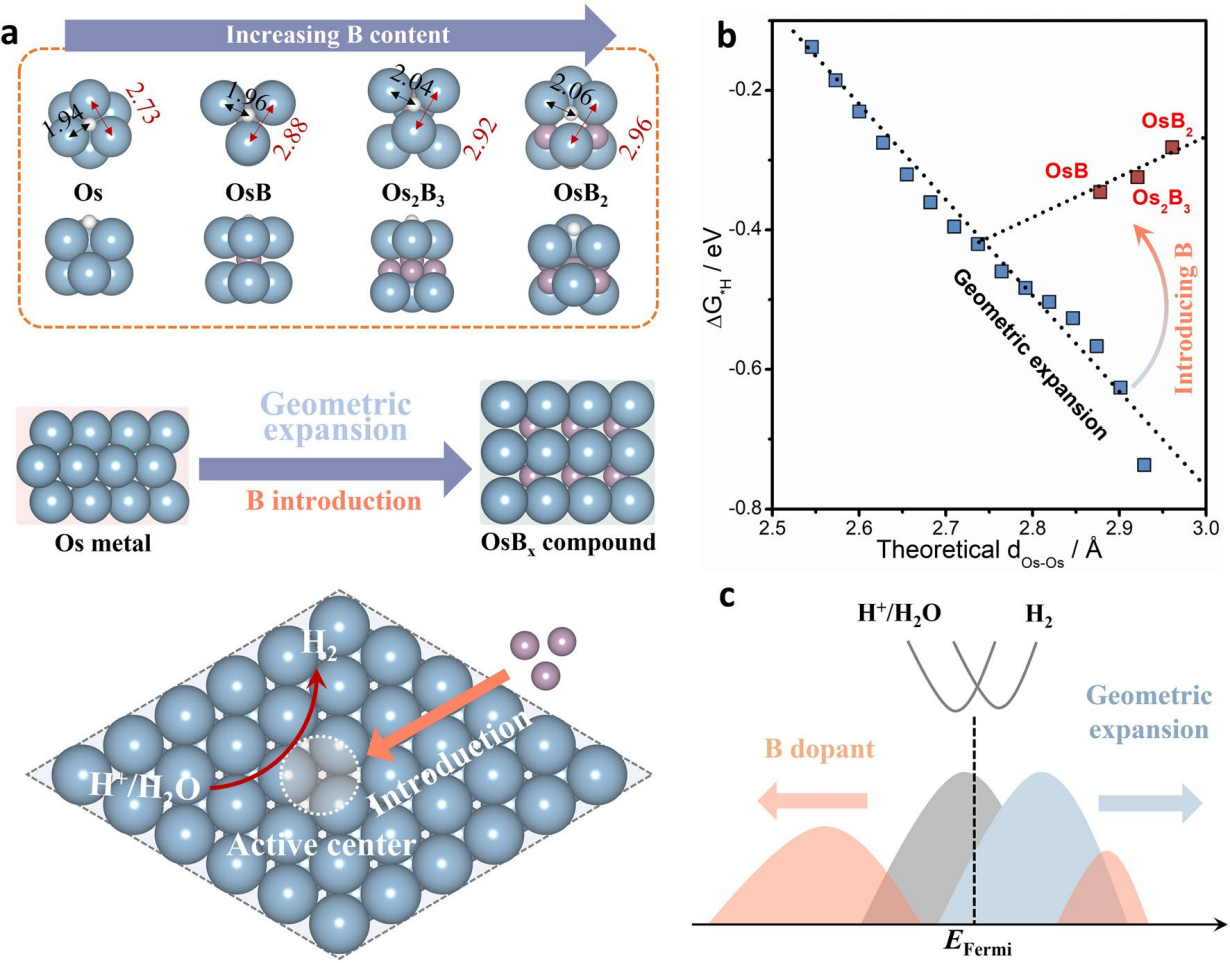
Theoretical calculations. **a** Atomic spacing modulation in intermetallic compounds for catalyzing HER. The purple, blue and white balls represent B, Os and H atoms, respectively. Top: the hydrogen adsorption center in Os, OsB, Os_2_B_3_, and OsB_2_. The black and red fonds denote the bonding length (Å) of Os-H and Os-Os, respectively. Middle: views of the change from Os metal to OsB_*x*_ intermetallic compounds. Bottom: schematic illustration of B-doping intermetallic compounds catalyzing HER. **b** The change in ΔG_*H_ along increased Os-Os bonding length. The arrow represents the effect of B introduction. **c** Schematic illustrating that the *d*-state shift induced by B dopant and geometric expansion

On the surface of metal catalysts, the hollow site (center of several atoms) usually displays strong H-binding state, serving as the active center to catalyze HER [23, 24]. Therefore, corresponding electronic structure optimization strategies are required to weaken the adsorption behavior of H intermediates. Different from the disorder and randomness of surface modification, we can find that when B with smaller atomic radius orderly occupies the interstitial sites of Os metals to form OsB_*x*_ (*x* = 1, 1.5, 2) intermetallic compounds, the volume effect caused by filling of B atoms is of gradient. As shown in Fig. 1a, with the increase in B contents in OsB_*x*_ intermetallic compounds, the d_Os-Os_ gradually increases from 2.73 to 2.96 Å, further leading to increase in Os-H bonding length in the hollow site (1.94–2.06 Å). Consequently, the transition of Os metals to OsB_*x*_ intermetallic compounds caused by the directional introduction of B geometrically expands the active center and furthers tune the conversion efficiency from H^+^/H_2_O to H_2_ by changing Os-H bonding length. Notably, the increase in the d_Os-Os_ during the conventional geometric expansion can induce the enhanced *H adsorption and a more sluggish kinetics in HER processes. Contrary to this, B-ordered interstitials achieve a reversal of the *H adsorption energy (ΔG_*H_) while maintaining a similar growth trend of the d_Os-Os_, which undoubtedly induces higher HER activity of OsB_*x*_ intermetallic compounds, especially OsB_2_. (Figs. 1b and S1). From the *d*-band theory (Fig. 1c), the geometric expansion usually leads to the weakening of Os-Os interaction and bring about the upshifted *ε*_d_ and enhanced adsorption ability. However, in addition to the volume effect, interstitial B atoms can induce *d*-band state splitting and downshift through the *s*, *p*-*d* orbital hybridization of new generated B-Os bonding. This further explains the possible reasons for the formation of this reversal trend. The above theoretical analysis reveals the feasibility of interstitial B to gradient disperse active site of Os to improve HER activity, which points out the direction for the subsequent synthesis of catalysts and the determination of the structure–activity relationship.

### Synthesis and Characterization of Catalysts

**Fig. 2:**
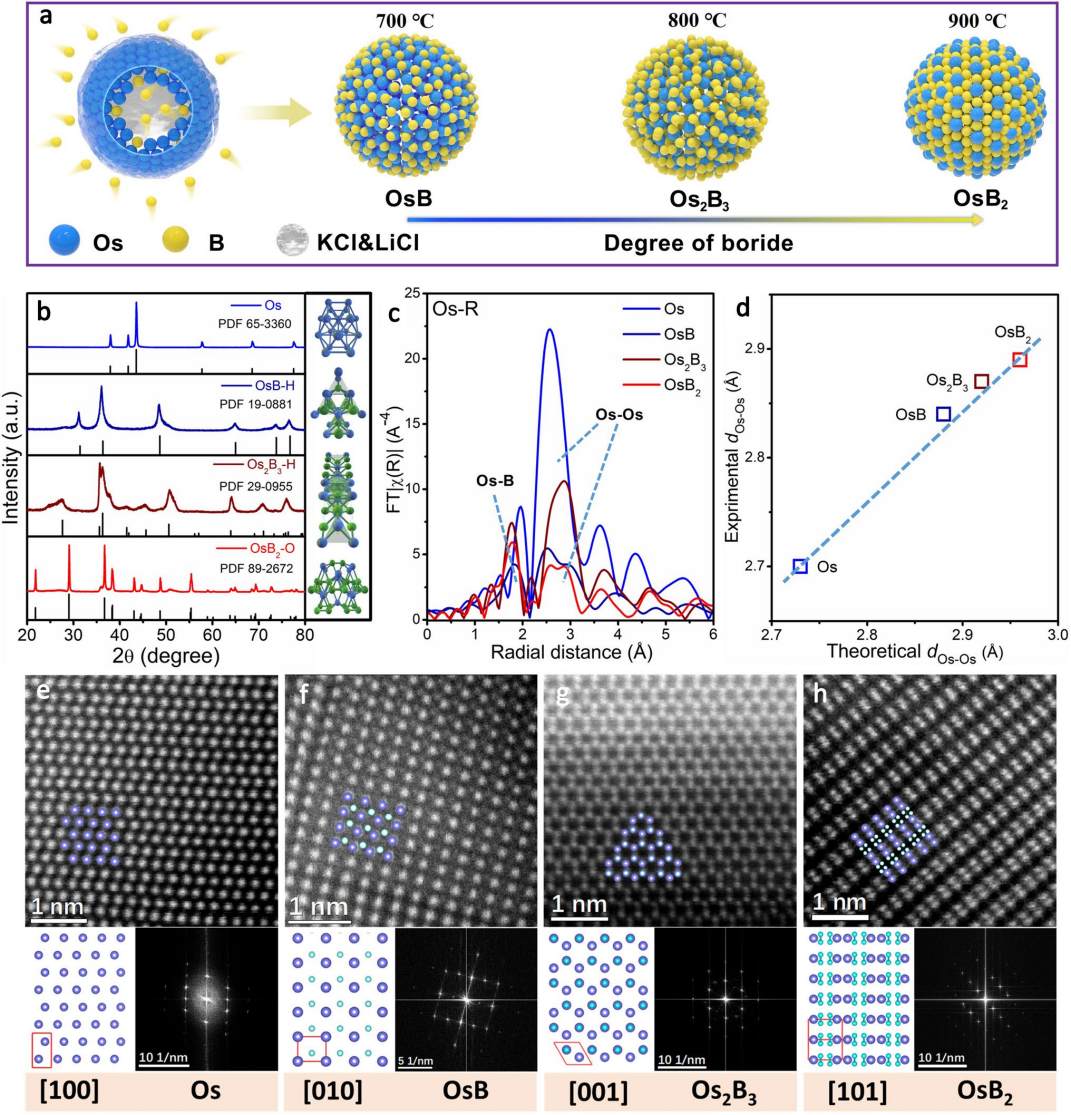
Structural characterizations**. a** Schematic illustration of the preparation of OsB_*x*_. **b** XRD patterns of all synthesized materials. **c** Os L_3_-edge EXAFS spectra of Os, OsB, Os_2_B_3_ and OsB_2_. **d** Linear relationship between theoretical and experimental Os-Os distance. High-resolution HAADF-STEM images and corresponding crystal structures and FFT patterns of **e** Os, **f** OsB, **g** Os_2_B_3_ and **h** OsB_2_, where Os atoms are in blue-violet while B atoms are in blue–white. (Color figure online)

Inspired by theoretical results, the one-pot molten salt-assisted route was conceived to construct OsB_*x*_ intermetallic compounds. As depicted in Fig. 2a, Os powder, excessive NaBH_4_ and KCl-LiCl eutectic prepared with lowest eutectic point (Fig. S2) were ground evenly and pyrolyzed to yield three kinds of intermetallic borides by controlling annealing temperature. Here the KCl-LiCl eutectic mixture provided a wide thermal stability window and negligible vapor pressure, which can enhance the atom diffusion, thus increasing the reaction rate and lowering the reaction temperature. Notably, both high temperature and liquid molten salt promote the interstitial filling process of B atoms, which is crucial for formation of ordered intermetallic compounds [25–28]. XRD results show that the products obtained at 700, 800 and 900 °C are hexagonal OsB (OsB-H) and Os_2_B_3_ (Os_2_B_3_-H), orthorhombic OsB_2_ (OsB_2_-O), respectively (Figs. 2b and S3). The inductively coupled plasma-optical emission spectrometry (ICP-OES) test shows that the content of B in OsB, Os_2_B_3_ and OsB_2_ is 5.6, 8.4, and 11.6 wt%, respectively, which further supports the corresponding structure of as-prepared OsB_*x*_ (Table S2). The realization of the ordered and graded interstitial B plays a vital role in exploration of the relationship between the host metal atomic spacing and catalytic activity. Figure 2c exhibits four typical Os L_3_-edge EXAFS spectra of Os, OsB, Os_2_B_3_ and OsB_2_, indicating that the filling of interstitial B induces the formation of new Os-B bonds and the gradual increase in the d_Os-Os_. Figure 2d further depicts a linear fitting between experimental and theoretically obtained d_Os-Os_ with high *R*-square of 0.98, and the dispersion of host Os atoms caused by interstitial B filling can be confirmed. Double spherical AC-STEM images with EDX elemental mapping (Figs. S4–S8) display a uniform distribution of Os and B in the Os, OsB, Os_2_B_3_ and OsB_2_ nanoparticles, respectively. In the STEM mode, the variation of the Os/B ratio can be resolved on an atomic scale. As shown in Fig. 2e–h, the well-resolved lattice atomic images and corresponding fast Fourier transform (FFT) patterns along the Os [100], OsB-H [010], Os_2_B_3_-H [001] and OsB_2_-O [101] zone axis match very well with the projected crystal structures. The above results fully prove that the synthesis of a series of ordered intermetallic borides successfully realizes the gradient dispersion of Os metal atoms.

### Electrochemical Evaluation toward HER

**Fig. 3:**
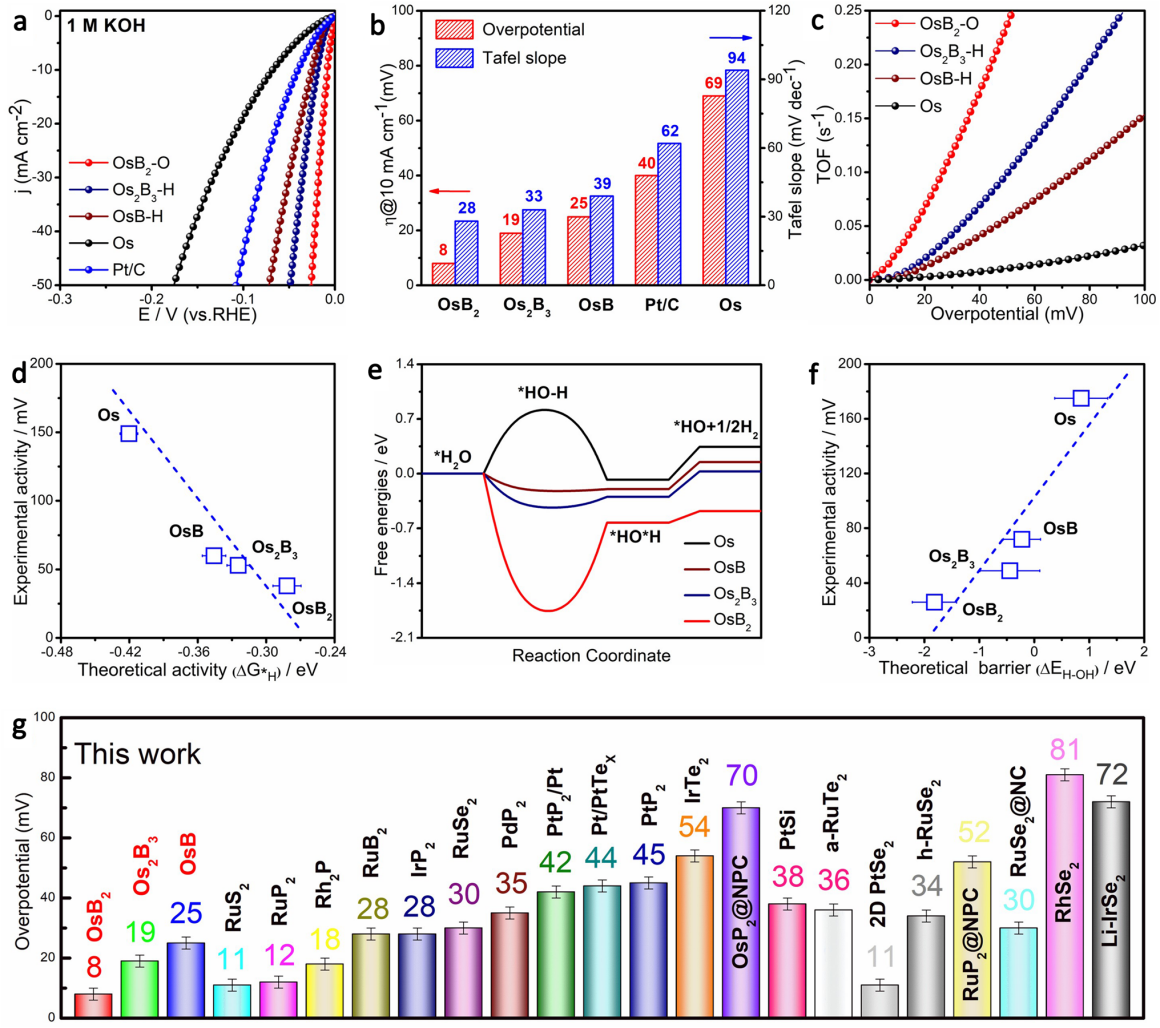
Apparent HER activity. **a** HER polarization curves, **b** corresponding overpotentials at 10 mA cm^−2^ and Tafel slope of Os, OsB_*x*_ and Pt/C in 1 M KOH. **c** The relationship between TOF and the measured potentials for Os and OsB_*x*_. **d** The correlation between theoretical and experimental activities in acid condition. **e** Free energy diagram along alkaline HER. **f** The correlation between energy barrier of H-OH splitting and experimental activities in alkaline environment. **g** Comparison of HER performance of OsB_*x*_ with recently reported Pt-group metal electrocatalysts at 10 mA cm^−2^ in 1 M KOH

Theoretical calculations and structural characterizations have shown that the d_Os-Os_ can be finely-tuned by filling the interstitial B to form different intermetallic borides. Next, we combined the electrochemical test and further DFT analysis to determine the correlation between electrocatalytic HER activity of catalysts and spacing modulation. The HER performance of pure Os, OsB_*x*_ and commercial Pt/C in 1 M KOH and 0.5 M H_2_SO_4_ was all evaluated in detail considering the different reaction mechanism in alkaline and acid media. Polarization (Fig. 3a) and Tafel (Fig. S9) curves demonstrate the HER activity and kinetics of OsB_*x*_ in 1 M KOH are better than those of Os and commercial Pt/C. Specifically, the operating overpotentials (@10 mA cm^−2^) follow the order: OsB_2_ (8 mV) < Os_2_B_3_ (19 mV) < OsB (25 mV) < Pt/C (40 mV) < Os (69 mV), and OsB_2_ possesses the lowest Tafel slope (28 mV dec^−1^) and HER pathways follow the Volmer–Tafel mechanism (Fig. 3b) [29–32]. Moreover, as shown in Fig. 3c, OsB_2_ delivers the highest TOF, which is 4.0-fold and 2.4-fold relative to Os_2_B_3_ and OsB at an overpotential of 50 mV. Thus, combining Tafel slope and TOF, it is proved that OsB_2_ possesses the best HER intrinsic catalytic activity, and the activity of OsB_*x*_ is positively correlated with d_Os-Os_. In addition, the *C*_dl_ and EIS test results also show the highest electrochemically active surface area (ECSA) and the smallest charge-transfer resistance (*R*_ct_) for OsB_2_ (Figs. S10–S11; Table S3), further elucidating the HER activity trend from Os, OsB, Os_2_B_3_ to OsB_2_. Similarly, we also probed the acidic HER performance on different samples by polarization curve, Tafel slope, TOF, *C*_dl_ and EIS (Figs. S12–S13). Os, OsB, Os_2_B_3_ and OsB_2_ still exhibit the same trend of HER activity as that in alkaline media, which further validates that the HER activity is enhanced with the increasing spacing of active Os atoms caused by the gradual filling of B atoms. Moreover, the HER polarization curves normalized to ECSA (Fig. S14) also indicate that OsB_2_ possesses excellent catalytic performance compared to Os, OsB, Os_2_B_3_ in 1 M KOH and 0.5 M H_2_SO_4_.

In addition, we first established a linear relationship between the measured HER performance (@50 mA cm^−2^) in acidic media and the ΔG_*H_ as a key electron transfer step for acidic HER (Figs. 3d and S15–S16). The good fitting relationship indicates that the weakening *H adsorption induced by the conversion of Os to OsB_2_ is one of the main reasons for promotion of HER activity. Comparably, alkaline HER undergoes the H_2_O dissociation except for *H adsorption and H–H coupling [33–35] it can be seen that on Os sites of Os, OsB, Os_2_B_3_ and OsB_2_, the H_2_O dissociation is the rate-potential step (RDS), limiting the process of HER (Fig. 3e). While, the decreased energy barrier with increasing B further provides faster HER kinetics. We further fitted the energy barrier and alkaline HER activity at 50 mA cm^−2^ (Fig. 3f), a good linear relationship certifies that interstitial B atoms accelerate the H_2_O dissociation, consequently improving the alkaline HER activity. In conclusion, the weakening *H adsorption and accelerated H_2_O dissociation due to the introduction of B atoms and the increase in d_Os-Os_ are the fundamental reasons for the enhanced activity of OsB_*x*_. Obviously, OsB_2_ is the optimal catalysts among our samples, which endows much better HER activity than most of state-of-the-arts Pt-group metal electrocatalysts especially in 1 M KOH (Fig. 3g; Table S4).

### Mechanism of Enhanced Activity and Stability

**Fig. 4:**
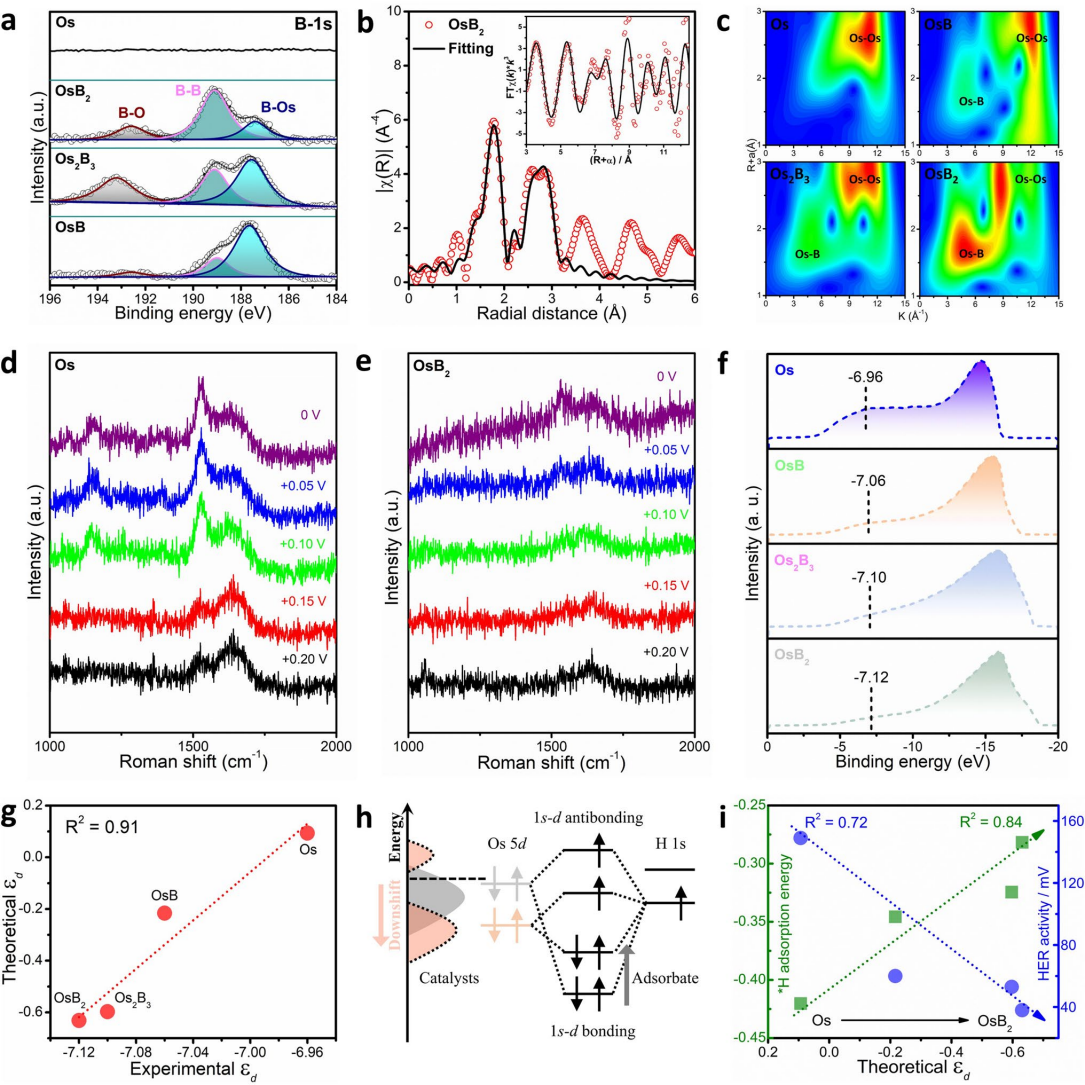
Mechanism of activity improvement. **a** XPS spectra of B 1*s* for corresponding products. **b** Os L_3_-edge EXAFS fitting curve of OsB_2_. **c** WT for the EXAFS signals. In situ Raman analysis of **d** Os and **e** OsB_2_. **f** UPS valence-band spectra of Os, OsB, Os_2_B_3_, and OsB_2_ relative to the Fermi level. **g** the correction between experimental and theoretical *ε*_d_. **h** The interaction of the *H *s*-orbital with the Os 5*d*-orbital. **i** Relationship of theoretical *ε*_d_, ΔG_*H_ and HER activity

First, the XPS survey patterns show the surface elemental composition of Os, OsB, Os_2_B_3_ and OsB_2_ (Fig. S17). Further combination of three emerging signal peaks assigned to B-O, B-B, and B-Os in the high-resolution spectrum of B 1*s* indicates the successful introduction of interstitial B (Fig. 4a) [36]. Meanwhile, relative to metal Os, the core level Os 4*f*_5/2_ and Os 4*f*_7/2_ of OsB_*x*_ is obviously shifted, revealing that the electronic structure of the active Os site was modulated (Fig. S18) [37, 38]. which can also be confirmed by the different Os L_3_-edge k^3^χ(k) oscillation spectra between Os and OsB_*x*_ (Fig. S19). Furthermore, the EXAFS coordination fitting results show that the ratios of Os-B and Os-Os coordination numbers (CN_Os-B_/CN_Os-Os_) in OsB, Os_2_B_3_, and OsB_2_ are 1.00, 1.44, and 2.06 (Figs. 4b and S20; Table S5), indicating that the richness of Os-B is improved and the interaction between host–guest elements is more intense in the process of ordered intercalation of B atoms. As shown in Fig. 4c, wavelet transform (WT)-EXAFS visualizes Os-B paths in OsB_*x*_. It has been discussed above that intercalation B can attenuate *H adsorption and accelerate H_2_O dissociation. Here, by comparing the broad peaks between 1500 and 1700 cm^−1^ in situ Raman spectra [39], it can be further demonstrated that the H_2_O adsorption of OsB_2_ is also significantly weakened relative to Os (Fig. 4d, e, the potential from + 0.20 to 0 V vs. RHE). Therefore, it is considered that the weakening adsorption of key intermediates in the HER pathway simultaneously optimizes both H_2_O dissociation and H binding, resulting in excellent HER activity of OsB_2_ in both acidic and alkaline media.

Next, we probed the occupied electronic states of Os and OsB_*x*_ catalysts by the UPS to understand the B-Os interaction on hydrogen adsorption ability. As shown in Fig. 4f, the measured *ε*_d_ for Os, OsB, Os_2_B_3_, and OsB_2_ are − 6.96, − 7.06, − 7.10, and − 7.12 eV, respectively. Therefore, the* p* − *d* hybridization between B and Os atoms causes the downshift of the *d* states for Os, making the negative shift of the *ε*_d_ for OsB_*x*_. Besides, we calculated the theoretical *ε*_d_ of Os, OsB, Os_2_B_3_, and OsB_2_ cells by means of DFT. And the good linear relationship between experimental and theoretical *ε*_d_ is displayed in Fig. 4g. This further certifies the downshift of the *ε*_d_ via B-Os interaction. The downshift of the *ε*_d_ weakens reactivity between Os 5*d* and H 1*s*, hence resulting in weak *H adsorption (Fig. 4h). This trend predicted by *d*-band theory also agrees well with the calculated *H adsorption (Fig. 4i). Namely, with the gradual filling of the interstitial B, the increase in d_Os-Os_ and enhancement of the interaction between the Os-B decrease the *ε*_d_, thereby weakening the H adsorption on the electrode surface during the HER process, and finally leading to a nearly linear increase in the catalytic activity. Besides, to further explain its relationship to the *d*-center position of Os, a quantitative analysis was conducted on the hybridization between B and Os (Fig. S21). It can be found that the decrease in Bader charge of B atoms resulted from the higher B-to-Os atomic ratio monotonically relates with the declining integrated crystal orbital Hamilton population (ICOHP) and the increase in Os *d*-band center, respectively. Therefore, we think the B charge state can simply quantify the *p*-*d* of hybridization between B and Os atoms, as a result of the balance of Os-Os, O-B and B-B interactions.

**Fig. 5:**
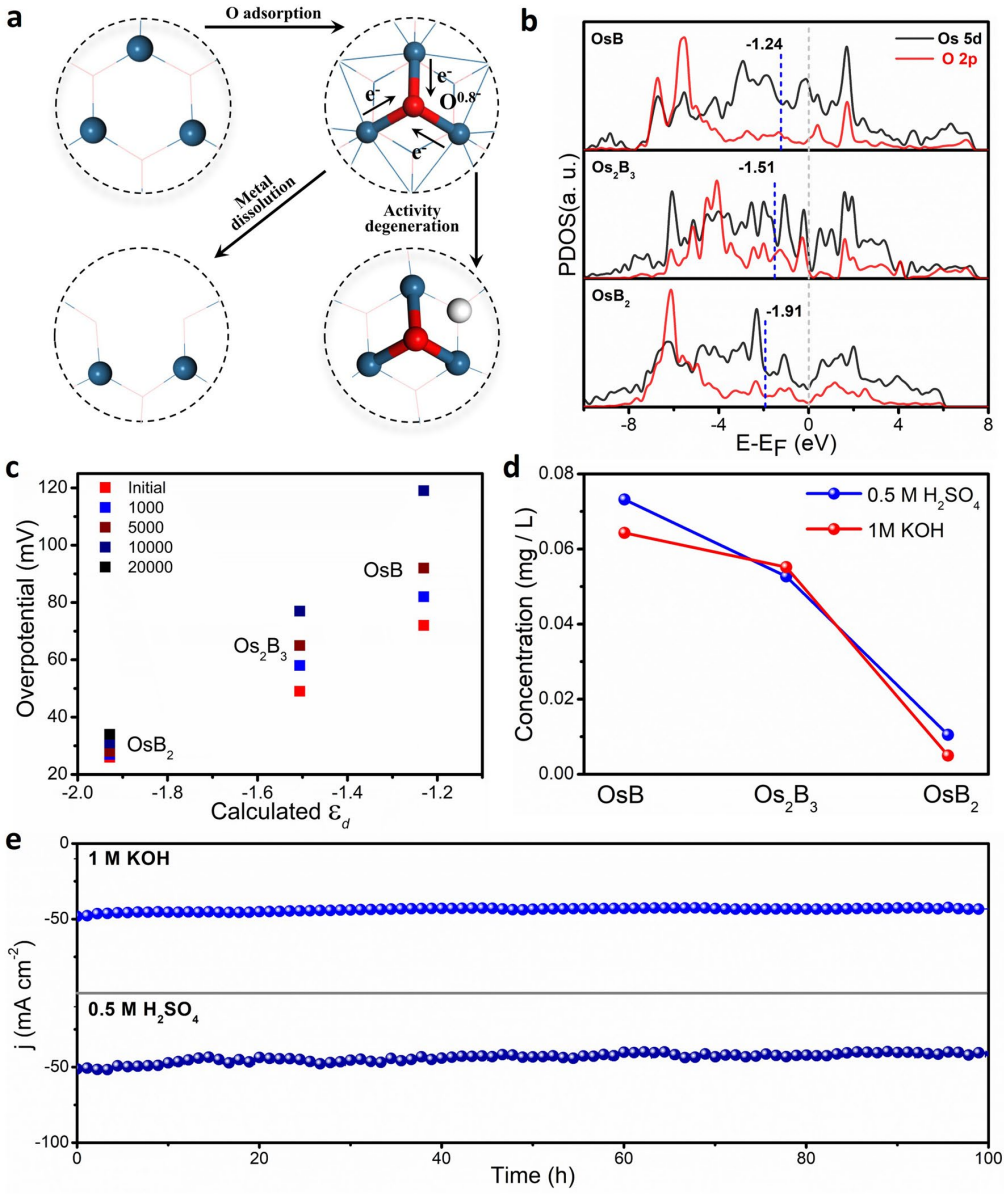
Mechanism of Stability improvement. **a** Two possible mechanisms of catalytic activity degeneration induced by strong O adsorption. **b** The PDOS calculations of O 2*p*- orbital and Os 5*d*-orbitals of relevant Os atoms and adsorbed O. **c** The relationship between the overpotential of the catalyst and ɛ_*d*_ after different acceleration cycles in 1 M KOH. **d** Concentration of Os in electrolyte dissolved from OsB, Os_2_B_3_ and OsB_2_ after electrocatalysis. **e** The operational durability of OsB_2_ in 1 M KOH and 0.5 M H_2_SO_4_

All HER polarization curves before and after different potential cycles in 1 M KOH and 0.5 M H_2_SO_4_ indicate that the stability of OsB_*x*_ was optimized with the increase in interstitial B content (Fig. S22). To find out why OsB_2_ is more stable, further intrinsic stability mechanisms were investigated. The stability of the electrode can be understood by the toxification and dissolution rate of the active site of catalysts [40]. As shown in Fig. 5a, the activity degradation of catalysts in the electrolyte may be due to two active failure paths caused by the formation of oxygenated species. One path is that the active site is strongly occupied, which further affects the adsorption ability of neighbor active centers. The other is that the adsorbed O causes the dissolution of the reactive metal Os. It can be found that the adsorbed O can obtain totally 0.8 e^−^ on all OsB_*x*_ compounds (Fig. S23). However, the surficial *ε*_d_ of Os atoms in active centers for OsB, Os_2_B_3_ and OsB_2_ is − 1.24, − 1.51 and − 1.91 eV, respectively (Fig. 5b). Based on the *d*-band theory, the upshifted *ε*_d_ via *O adsorption can induce the strengthen reactivity, resulting in the strong *H adsorption and HER performance degenerations. Meanwhile, the enhanced adsorption ability of Os can more easily interact with extra oxygenated species. With more binding O, the Os can gradually dissolve into electrolytes in the form of Os ion and OsO_*x*_^−^, which results in the loss of active sites. The relationship between the overpotential of the catalyst and the ɛ_*d*_ after different acceleration cycles (Figs. 5c and S24), and the concentration change of Os in electrolytes after electrocatalysis (Fig. 5d) further corroborates the above theoretical analysis. Therefore, the more complex the intermetallic boride structure and the stronger the coordination effect, the better anti-oxidative poisoning and dissolution stability are during the HER process. Indeed, OsB_2_ can maintain a stable operating current (Fig. 5e) during durability tests (100 h) in acidic and alkaline media, which is significantly better than that of commercial Pt/C (Figs. S25–S26). Besides, the crystal structure and the surface electronic state stability of OsB_2_ during the HER process are further confirmed by XRD, XPS, and TEM characterization (Figs. S27–S29).

## Conclusions

In summary, this work uncovers a novel control method over the atomic spacing of active metal sites (d_M-M_) through light interstitial atom filling, significantly enhancing the catalytic activity and stability in either acidic or alkaline HER. This is attributed to the fact that the interstitial atom (e.g., B) not only reduces the H_2_O dissociation barrier with increasing atomic spacing of active metal (e.g., Os) sites, but also induces the downshift of the ɛ_*d*_ through the strong correlation between active metal site-light atom (such as Os-B), thereby reversing the hydrogen adsorption-distance relation and optimizing the H binding on the electrode surface. The unity of theory and experiment fully confirms that the largest d_Os-Os_ (2.96 Å) is the most active HER catalyst among samples filled with different amount of B. Due to the most abundant Os-B coordination environment, it is conferred with the ability to effectively inhibit the inactivation and dissolution of active substances during HER. Our discovery demonstrates an efficient strategy for finely tuning atomic spacing and a reversed hydrogen adsorption-distance relationship, which are an important step forward toward clarifying structure–activity relationships at the atomic-level and developing advanced catalysts.

### Supplementary Information

Below is the link to the electronic supplementary material.

Supplementary file1 (PDF 2850 KB)
