# Supplementary material for: Tuning Active Metal Atomic Spacing by Filling of Light Atoms and Resulting Reversed Hydrogen Adsorption-Distance Relationship for Efficient Catalysis
Source: Nanomicro Lett. 2023 Jul 3;15:168. doi: 10.1007/s40820-023-01142-1 (PMC10317938; doi:10.1007/s40820-023-01142-1)
Supplement: Supplementary file 1 — Supplementary file1 (PDF 2850 KB) [file 40820_2023_1142_MOESM1_ESM.pdf]

Supporting Information for

Tuning Active Metal Atomic Spacing by Filling of Light Atoms and Resulting Reversed Hydrogen Adsorption-Distance Relationship for Efficient Catalysis

Ding Chen[1, ‡], Ruihu Lu[1, ‡], Ruohan Yu[2], Hongyu Zhao[1], Dulan Wu[1], Youtao Yao[1], Kesong Yu[1], Jiawei Zhu[1], Pengxia Ji[1], Zonghua Pu[1], Zongkui Kou[1], Jun Yu[1], Jinsong Wu[2], Shichun Mu[1, *]

[1]State Key Laboratory of Advanced Technology for Materials Synthesis and Processing, Wuhan University of Technology, Wuhan 430070, P. R. China

[2]NRC (Nanostructure Research Centre), Wuhan University of Technology, Wuhan 430070, P. R. China

[‡]Ding Chen and Ruihu Lu contributed equally to this work.

*Corresponding author. E-mail: msc@whut.edu.cn (Shichun Mu)

S1 Experimental Details

S1.1 Computational Details

We conducted spin-polarized DFT calculations using the Vienma ab initio simulation package (VASP) [S1, S2]. Using the electron exchange and correlation energy was modelled within the generalized gradient approximation in the Perdew−Burke−Ernzerhof functional (GGA-PBE) [S3], while the projector augmented ware (PAW) pseudo-potentials was used to describe ionic cores [S4]. The long-range dispersion interactions between adsorbates and surface were treated applying DFT-D3 method [S5] the calculations were done with a plane-wave basis set defined by a kinetic energy cutoff of 450 eV. The *k*-point sampling was obtained from the Monkhorst−Pack scheme with a (3 × 3 × 1) mesh for optimization and a (4 × 4 × 1) mesh for the calculations of electronic structure. The geometry optimization and energy calculation are finished when the electronic self-consistent iteration and force were reach 10^{-5} eV and 0.02 eV Å^{-1}, respectively. A 6-layer p(4×4) Os (001), 5-layer p(4×4) OsB (001), 1.5-layer p(3×3) Os_2B_3 (001), and 2-layer $cOsB_2$ (011) supercells were built within 15 Å vacuum layers. During optimization, we fixed the bottom 3 layers Os (001), 3-layers OsB (001), 0.5-layer Os_2B_3 (001), and 1l-layer OsB_2 (011), respectively, whilst the other layers were free for relaxation along z direction.

HER

The HER process is divide into the four fundamental reactions as following：

（1） $H^+ + e^- + * = H*$

（2） $H^* = * + 1/2\ H_2$

H^* presents the H moiety on the adsorption site. Where which the energy of H^+/e^- is approximately equal to the energy of $1/2\ H_2$ [S6].

The Gibbs Free Energy Variation

The change in Gibbs free energy (ΔG) of each adsorbed intermediate is calculated based on the computational hydrogen electrode method developed by Nørskov et al.8 At standard condition (T = 298.15 K, pH = 0, and U = 0 V (vs. SHE)), the free energy G is defined as the following equation:

$$\Delta G = \Delta E + \Delta E_{ZPE} - T\Delta S$$

Where ΔE is the energy change obtained from DFT calculation, ΔE_{ZPE} is the difference between the adsorbed state and gas, which was calculated by summing vibrational frequency for all model based on the equation: $E_{ZPE} = 1/2\sum h\nu_i$. T is the temperature (298.15 K) in the above reaction system, and ΔS represents the difference on the entropies between the adsorbed state and gas phase. The entropies of free molecules were obtained from NIST database (https://janaf.nist.gov/). And the free energy of the adsorbed state H* can be taken as: $\Delta G_H = \triangle E_{H^*} + 0.24$ [S6].

***d*-band center**

The *d*-band center proposed by Nørskov and co-workers [S7] is a semi-quantitative descriptor to describe the trend of reactivity of transition metals (TM), which is defined the *d*-band center (ε_d) relative to the Fermi level (E_F). A transition metal with a low ε_d value relative to the Fermi level, shows a weak adsorption for a given adsorbate. And the *d*-band center (ε_d) is calculated as following：

$$\varepsilon_d = \frac{\int_{-\infty}^{+\infty} x\rho(x)\mathrm{d}x}{\int_{-\infty}^{+\infty} \rho(x)\mathrm{d}x}$$

Where ρ(x) is the projector density of states (PDOS) with respect to Os sites in Os metal and OsB_x intermetallic compounds

S1.2 Materials and Reagents

Osmium (Os) powder was purchased from Aladdin Reagents Ltd. Anhydrous lithium chloride (LiCl) and potassium chloride (KCl), sodium borohydride ($NaBH_4$), potassium hydroxide (KOH), sulfuric acid (H_2SO_4), absolute ethanol and isopropyl alcohol were purchased from Sinopharm Chemical Reagent Co., Ltd. Commercial Pt/C (20 wt%) and Nafion (5 wt%) were obtained from Sigma-Aldrich. All the reagents are analytical grade and used without further treatment. Deionized (DI) water was employed as solvent.

S2 Supplementary Figures and Tables

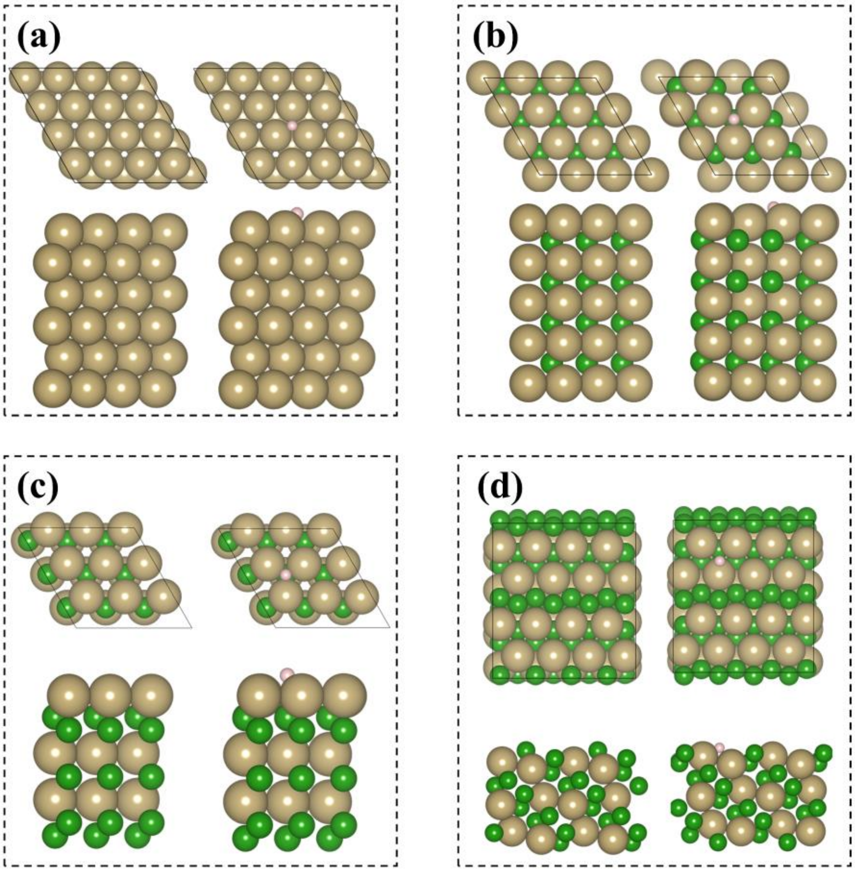

Fig. S1 Top and side views of Os, OsB, Os_2B_3, and OsB_2 in pristine models and models with *H adsorption

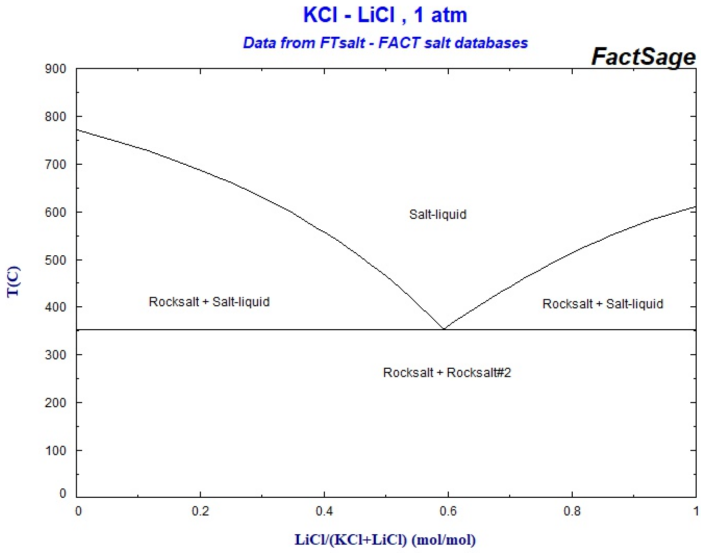

Fig. S2 The phase diagram of the two-salt system (KCl + LiCl), which coming from the http://www.crct.polymtl.ca/FACT/documentation/ (FTsalt → KCl-LiCl)

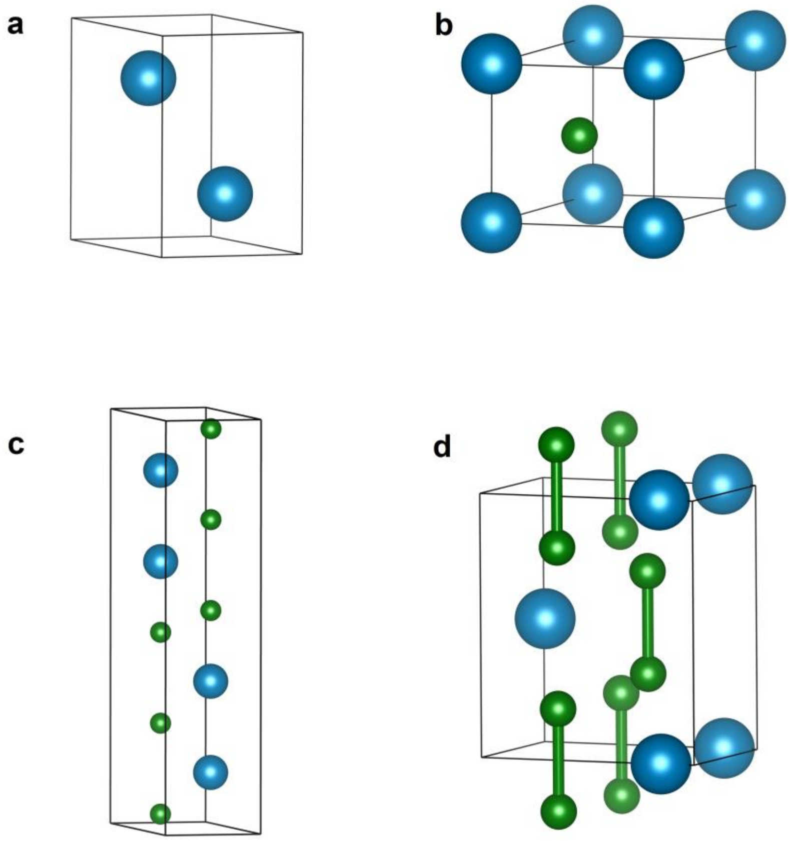

Fig. S3 Crystal structure (unit cell) of Os (**a**), hexagonal phase OsB (**b**), hexagonal phase Os_2B_3 (**c**) and orthorhombic phase OsB_2 (**d**)

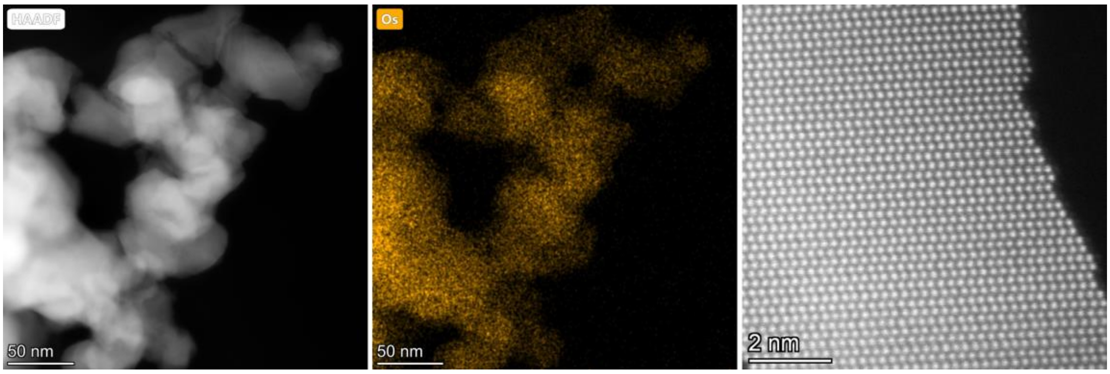

Fig. S4 HAADF-STEM images and corresponding EDX elemental map for Os of metal Os

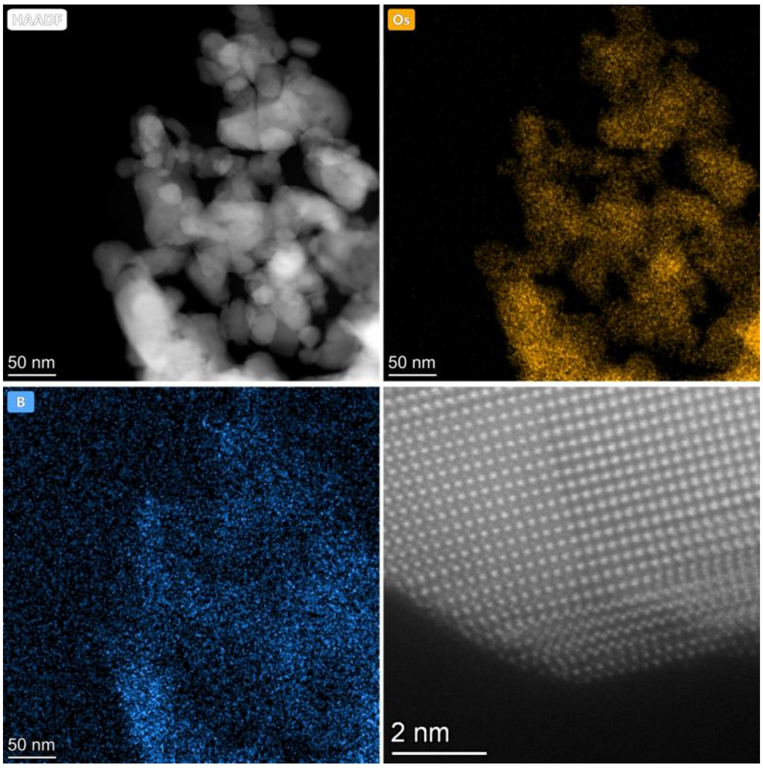

Fig. S5 HAADF-STEM images and corresponding EDX elemental maps for Os and B of OsB

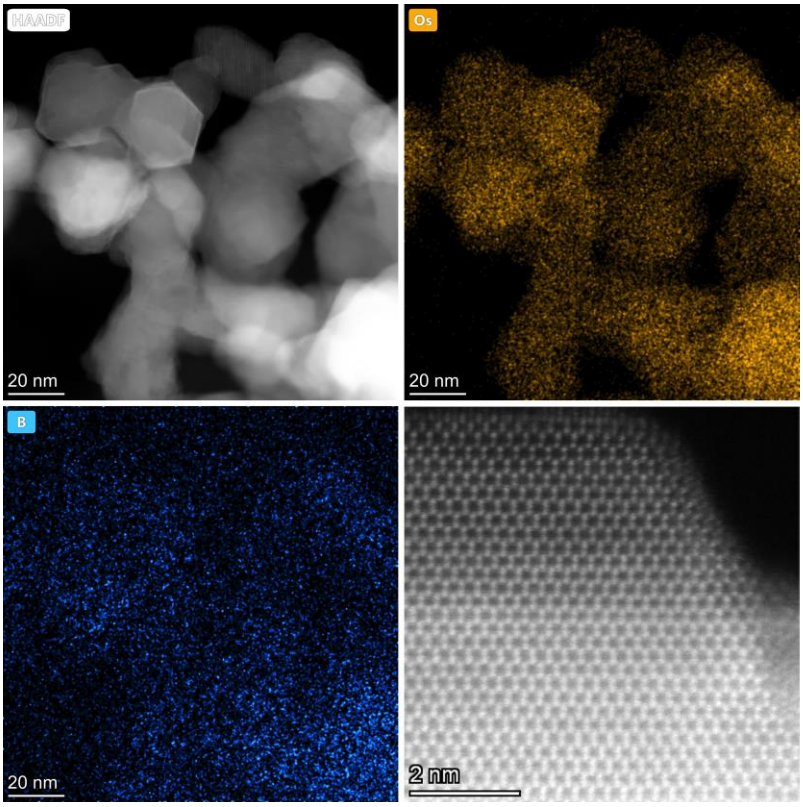

Fig. S6 HAADF-STEM images and corresponding EDX elemental maps for Os and B of Os_2B_3

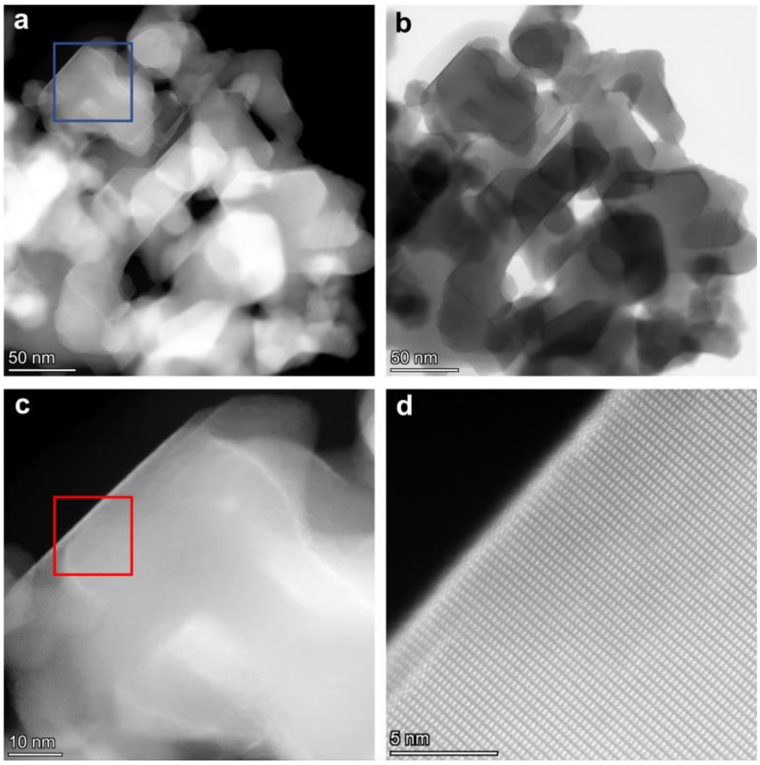

Fig. S7 (**a, b**) HAADF and corresponding BF images of OsB_2. (**c**) Enlarged STEM image from the area indicated by the blue box in (**a**). (**d**) Corresponding high-resolution atomic image from the area indicated by the red box in (**c**)

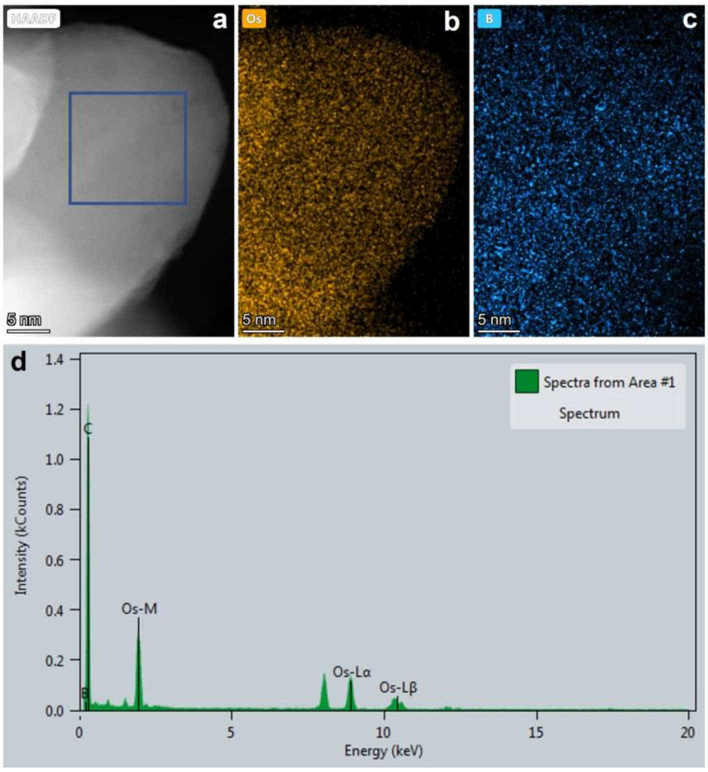

Fig. S8 (**a-c**) HAADF-STEM image and corresponding EDX elemental maps for Os and B of OsB_2. (**d**) EDX spectrum of the area indicated by the blue box in (**a**)

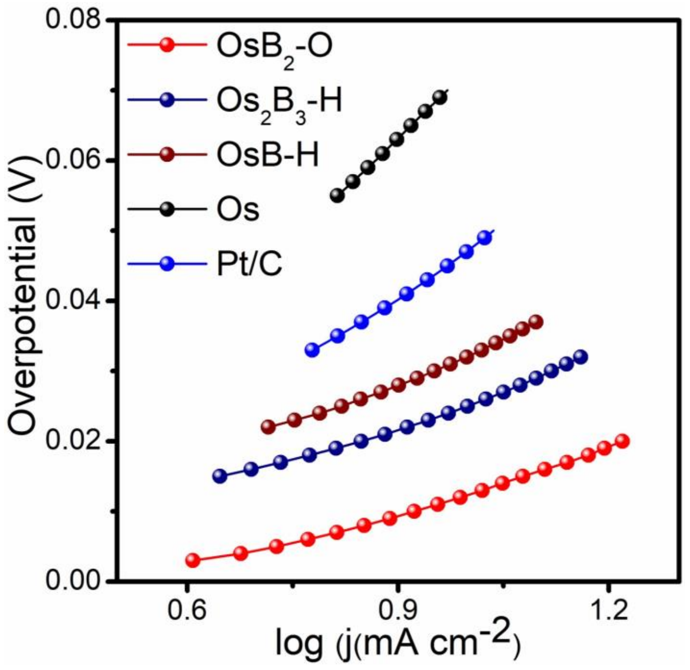

Fig. S9 Tafel curves of Os, OsB_X and Pt/C in 1 M KOH

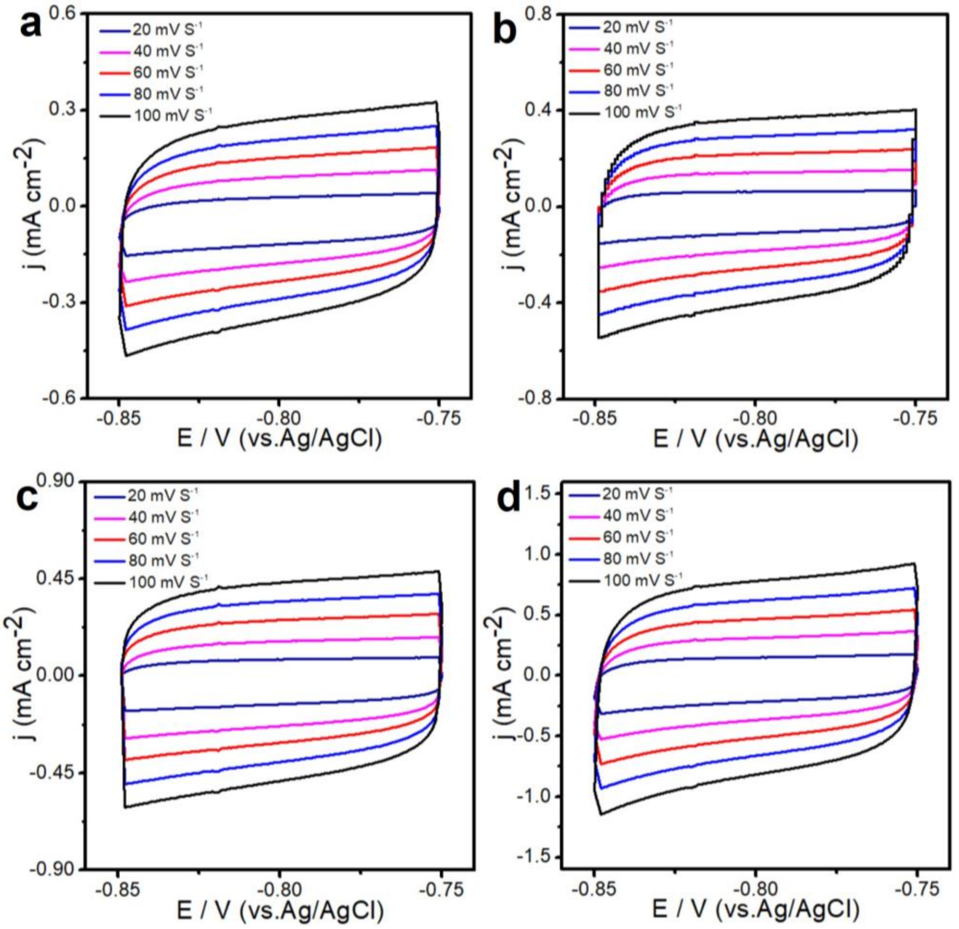

Fig. S10 Cyclic voltammograms of (**a**) Os, (**b**) OsB, (**c**) Os_2B_3 and (**d**) OsB_2 in the region of (-0.85) - (0.75) V versus Ag/AgCl at different scan rates

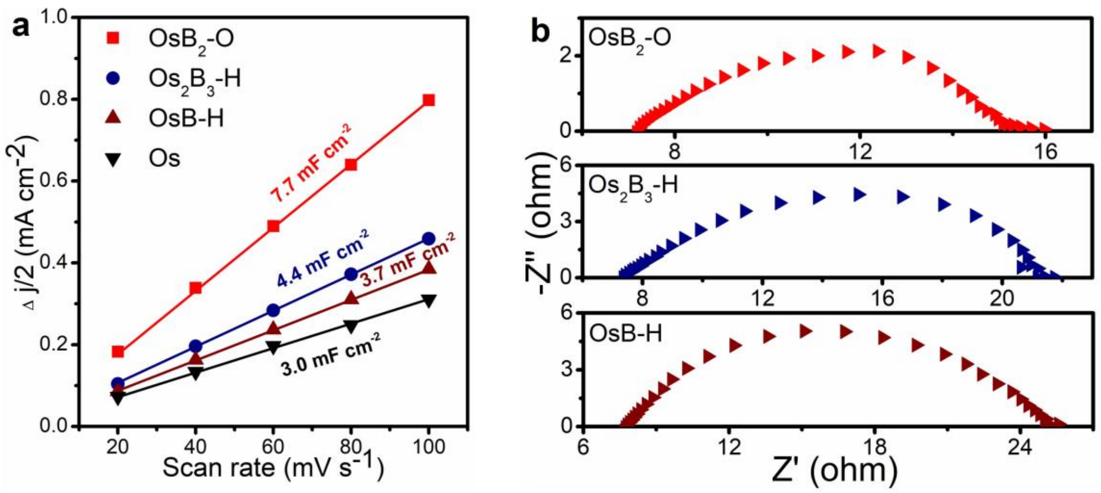

Fig. S11 (**a**) Linear relationships between capacitive current and scan rate of Os and OsB_X. (**b**) Nyquist plots of OsB, Os_2B_3 and OsB_2

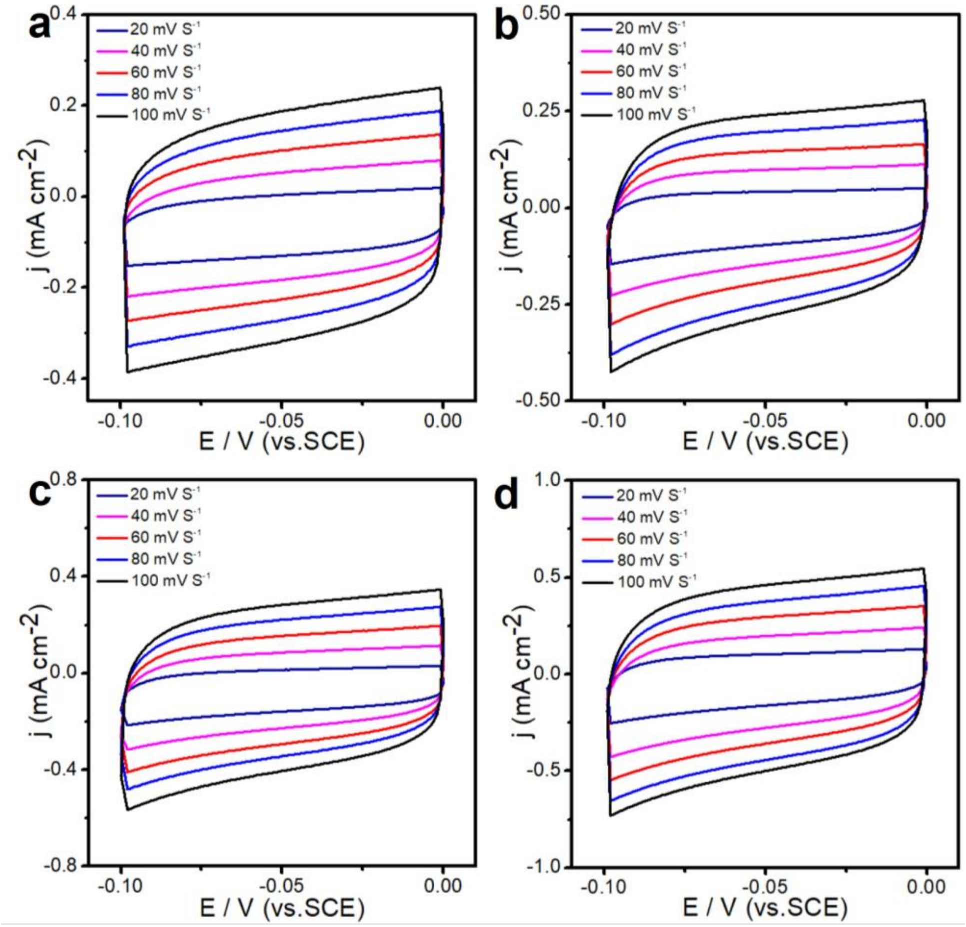

Fig. S12 Cyclic voltammograms of (**a**) Os, (**b**) OsB, (**c**) Os_2B_3 and (**d**) OsB_2 in the region of (-0.1) - (0.0) V versus SCE at different scan rates

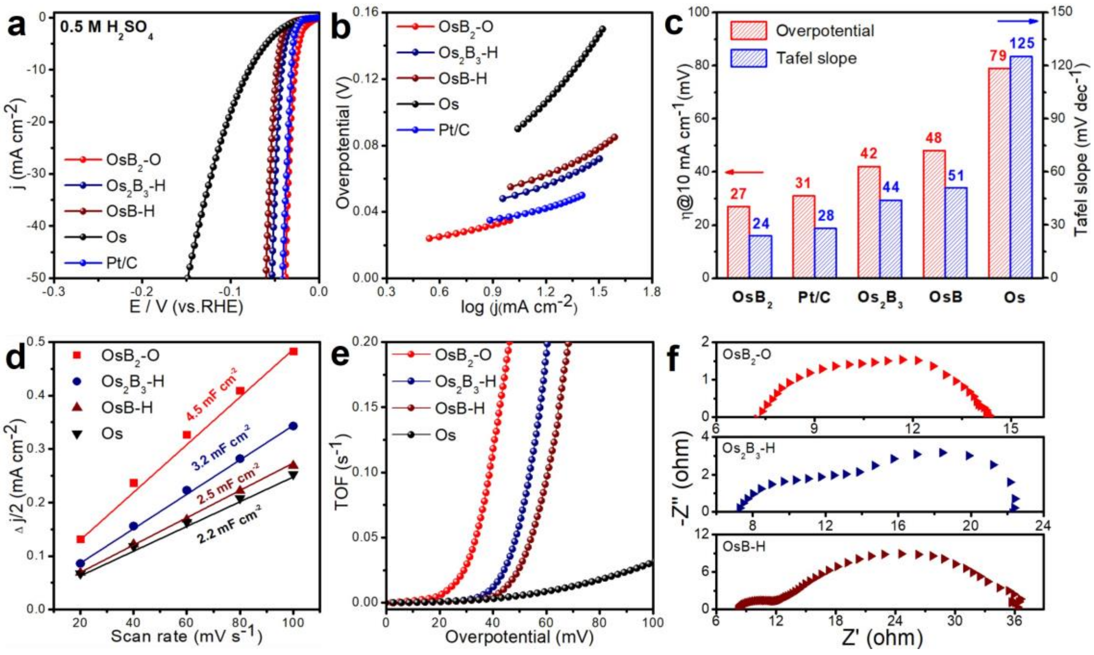

Fig. S13 Evaluation of HER performance in 0.5 M H_2SO_4. (**a**) HER polarization curves, (**b**) Tafel curves, (**c**) corresponding overpotentials at 10 mA cm^{-2} and Tafel slope of Os, OsB_X and Pt/C. (**d**) Linear relationships between capacitive current and scan rate, (**e**) the relationship between TOF and the measured potentials for Os and OsB_X. (**f**) Nyquist plots of OsB, Os_2B_3 and OsB_2

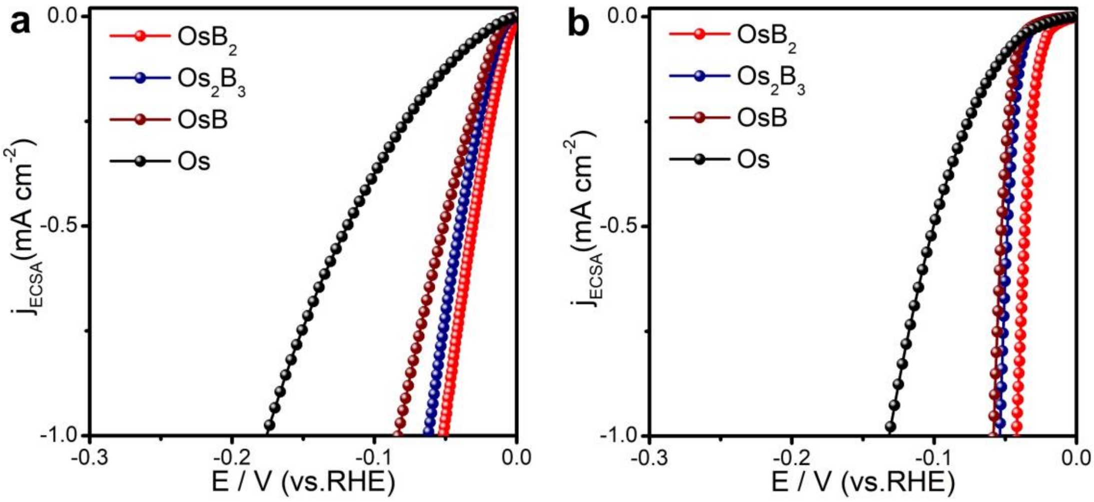

Fig. S14 LSVs normalized by ECSA of HER in 1 M KOH (**a**) and 0.5 M H_2SO_4 (**b**)

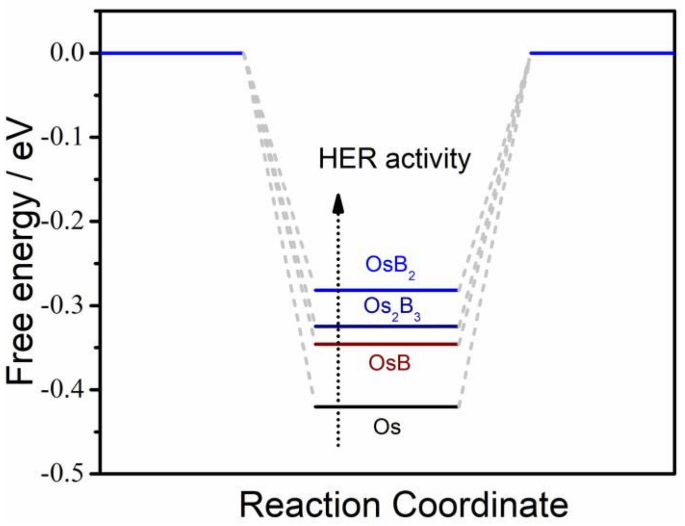

Fig. S15 Gibbs free energies changes of HER process on Os sites of Os, OsB, Os_2B_3 and OsB_2

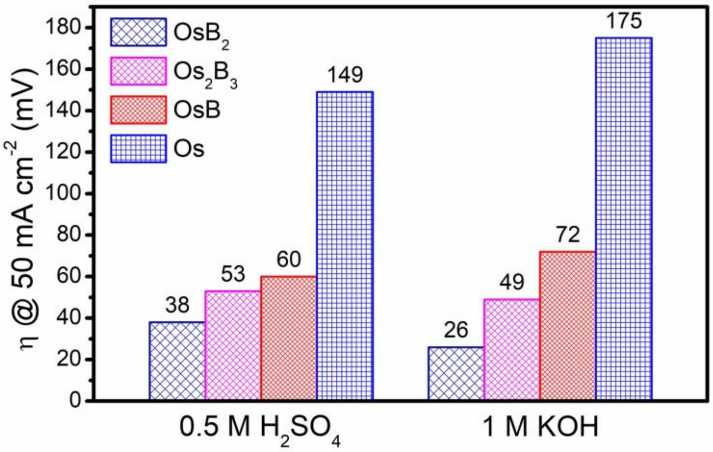

Fig. S16 HER overpotentials (@50 mA cm^{-2}) of Os, OsB, Os_2B_3 and OsB_2 in 0.5 M H_2SO_4 and 1 M KOH, respectively

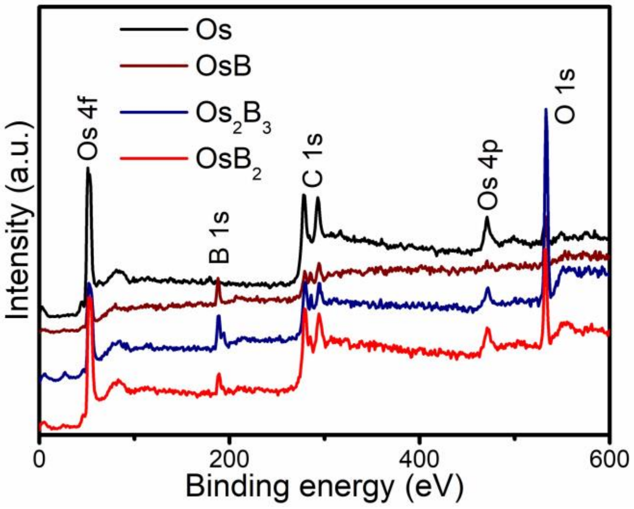

Fig. S17 XPS survey patterns of Os, OsB, Os_2B_3 and OsB_2

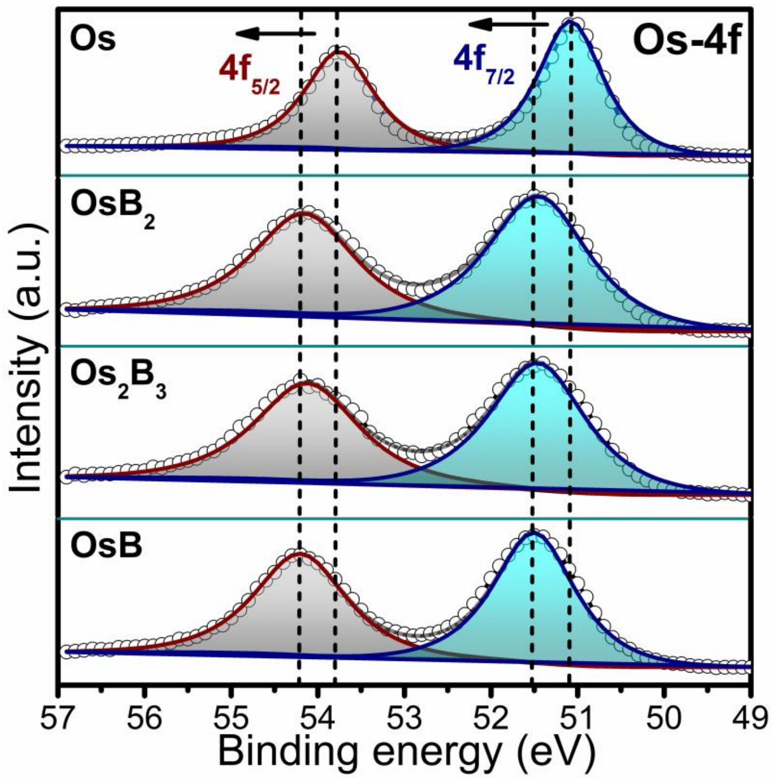

Fig. S18 XPS spectra of Os 4f for Os, OsB, Os_2B_3 and OsB_2

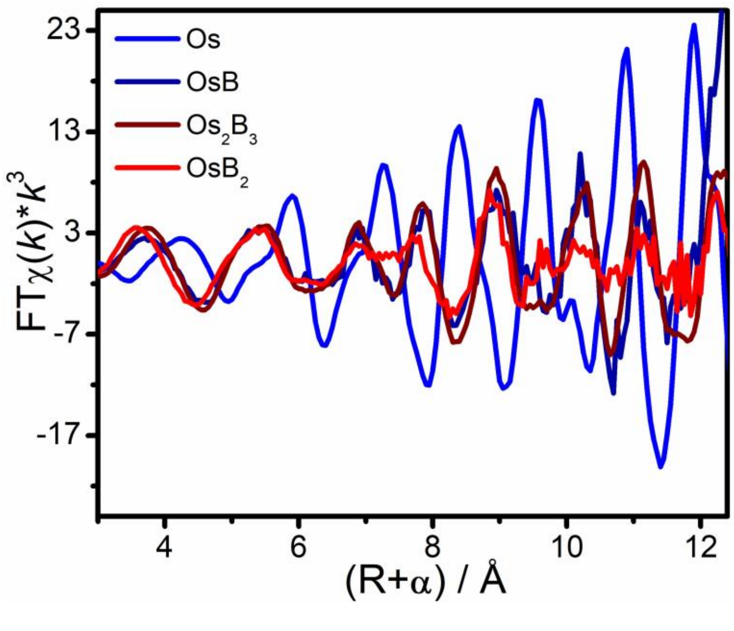

Fig. S19 Os *K*-space oscillation, weighted by k^3

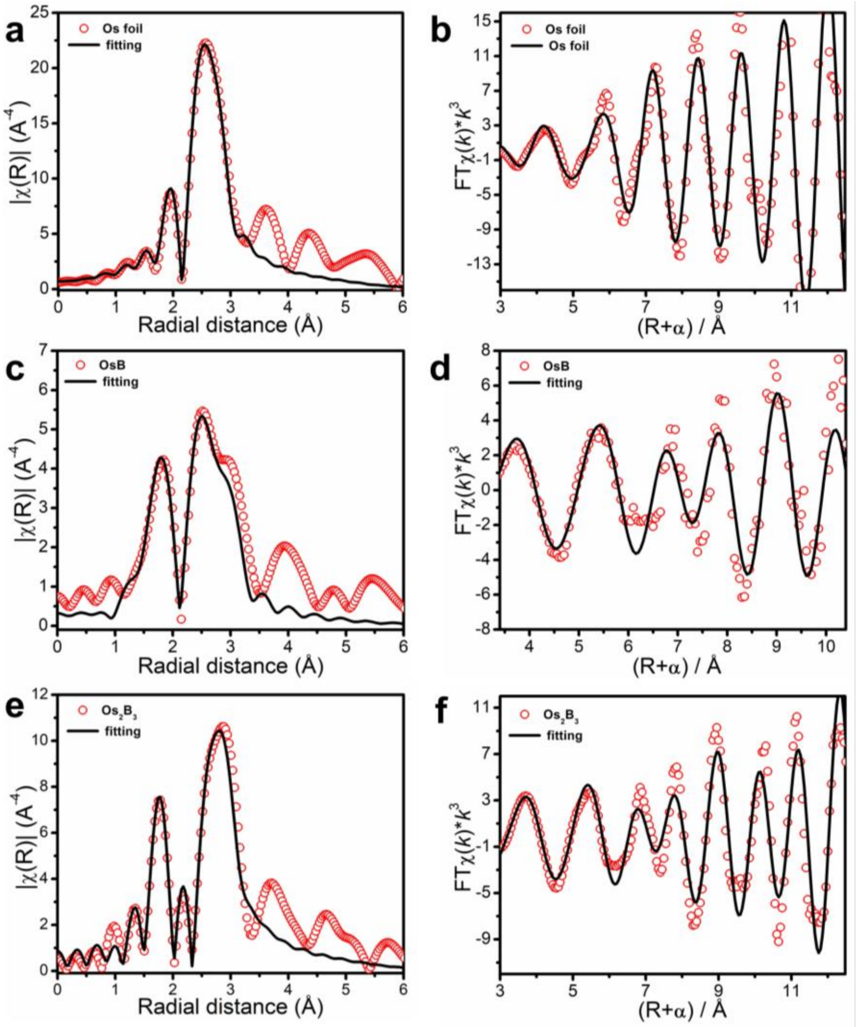

Fig. S20 The χ(R) and χ(k) space spectra fitting curves of Os L_3-edge for Os (**a, b**), OsB (**c, d**), Os_2B_3 (**e, f**)

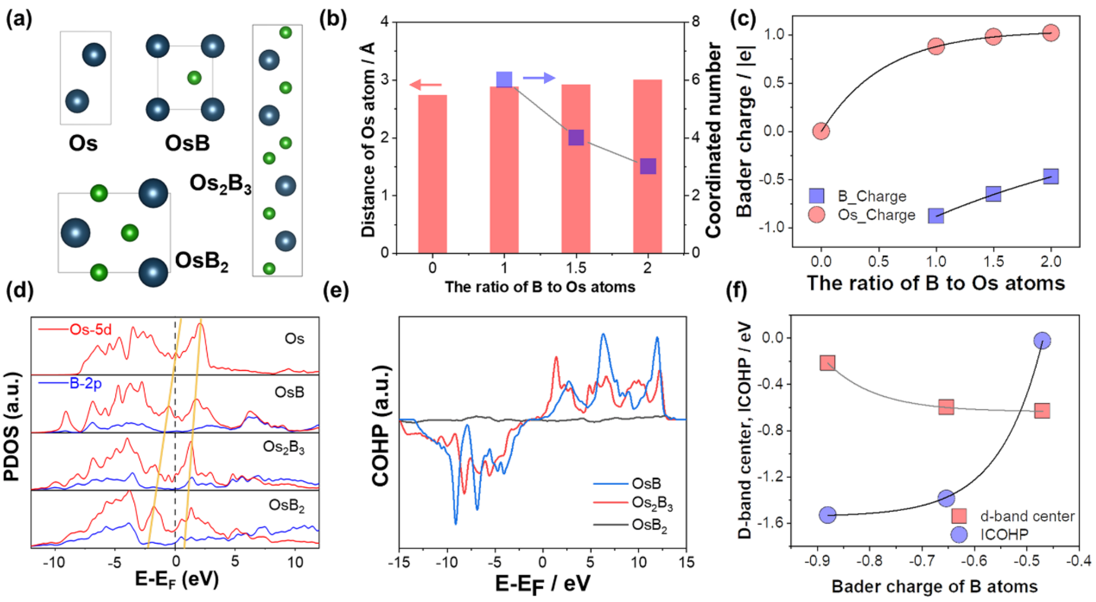

Fig. S21 (**a**) Models of Os, OsB, Os_2B_3, and OsB_2 cell. The green and indigo balls

represent B, Os atoms, respectively. (**b**) Correlations between surface Os-Os distance, B coordinated number and the ratio of B to Os atoms in Os, OsB, Os_2B_3, and OsB_2 cell. (**c**) Trends of Bader charge of B and Os atoms relative to the ratio of B to Os atoms in Os, OsB, Os_2B_3, and OsB_2 cell. (**d**) Projector density of states of B-2*p* and Os-5*d* orbitals. (**e**) COHP between B and Os atoms. (**f**) Correlation between *d*-band center, ICOHP and Bader charge of B atoms

Description: We first optimized the Os, OsB, Os_2B_3, and OsB_2 cell in **Fig. S21a**. We found the surface Os-Os distance increases while bulk B coordinated number decreases as the ratio of B to Os atoms increases, as displayed in **Fig. S21b**, underlyingly suggesting the weakening of Os-Os and Os-B interactions. Besides, the incremental B-to-Os ratio leads to more B atoms share the electrons from Os atoms, showing the more positive charge states of Os atoms and less negative charge states of B atoms in **Fig. S21c**. It points to that Os-B interaction replaces Os-Os interaction to stabilize Os atoms and more B-B interaction lows the strong *p*-*d* hybridization between B and Os. Accordingly, as shown in **Fig. S21d**, the originally metallic anti-bonding states above the Fermi level gradually shift to the lower level while the weaker Os-B interactions impedes the upshift of Os 5*d*-bands, totally meaning the downshift of Os band, in well agreement with the decrease of bonding states in **Fig. S21e**. therefore, the Bader charge of B atoms can be a simple descriptor to describe the change of Os 5*d*-bands in **Fig. S21f**. Furthermore, we found that the decrease of Bader charge of B atoms resulted from the higher B-to-Os atomic ratio monotonically relates with the declining integrated crystal orbital Hamilton population (ICOHP) and increase of Os *d*-band center, respectively.

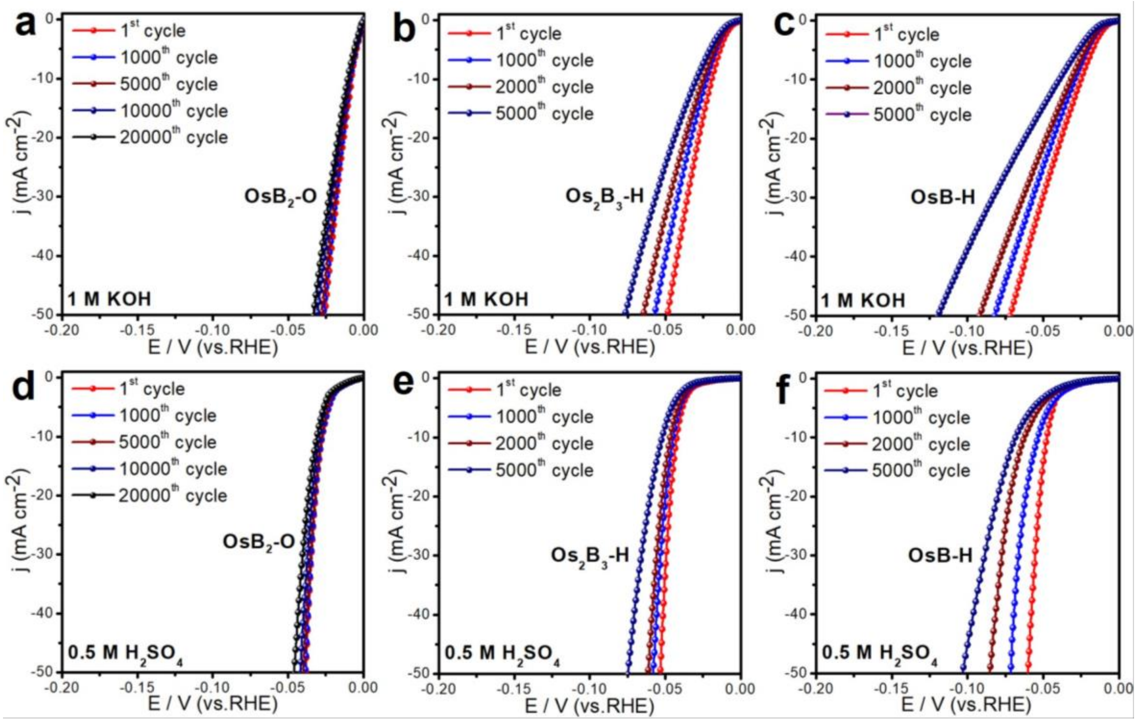

Fig. S22 HER polarization curves before and after different potential cycles in 1 M KOH and 0.5 M H_2SO_4 of OsB_2 (**a, d**), Os_2B_3 (**b, e**) and OsB (**c, f**)

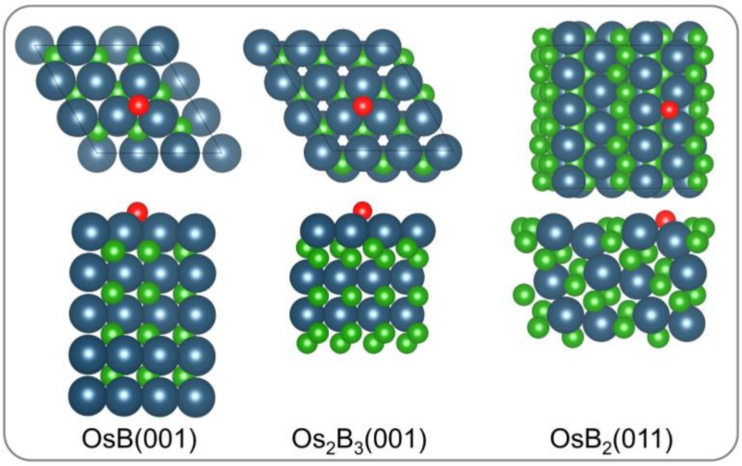

Fig. S23 Top and side views of *O adsorbed OsB (001), Os_2B_3 (001) and OsB_2 (011) models. The green, red, and indigo balls represent B, O, and Os atoms, respectively

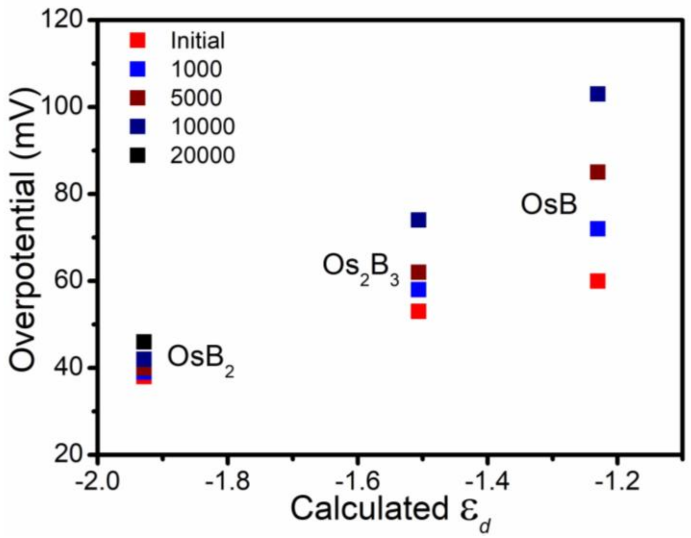

Fig. S24 The relationship between the overpotential of the catalyst and the ε_d after different acceleration cycles in 0.5 M H_2SO_4

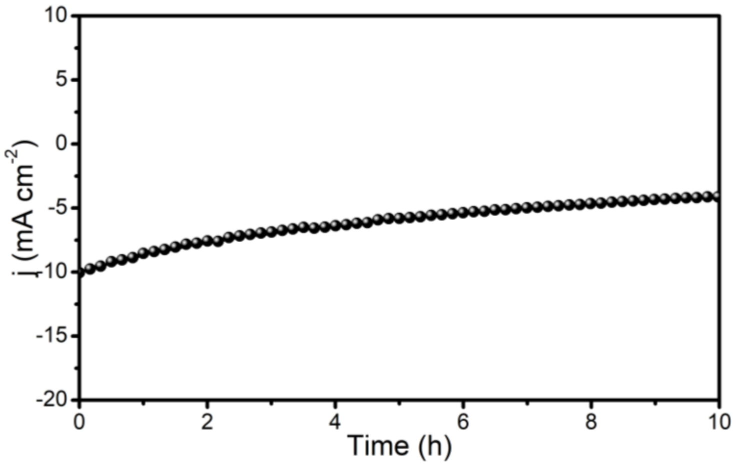

Fig. S25 Time-dependent current density curve of Pt/C in 1 M KOH

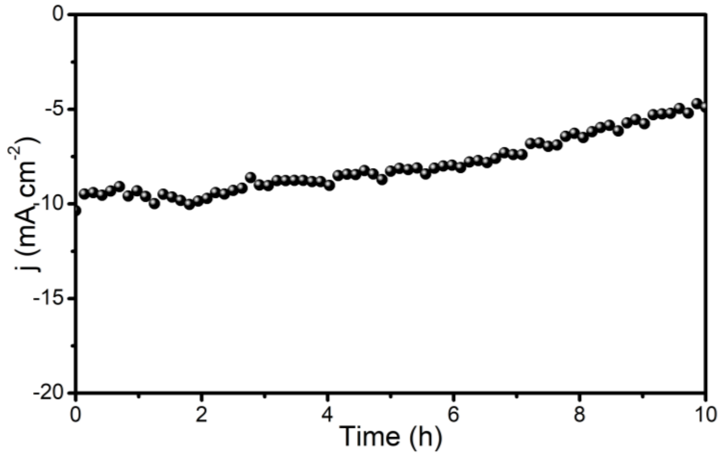

Fig. S26 Time-dependent current density curve of Pt/C in 0.5 M H_2SO_4

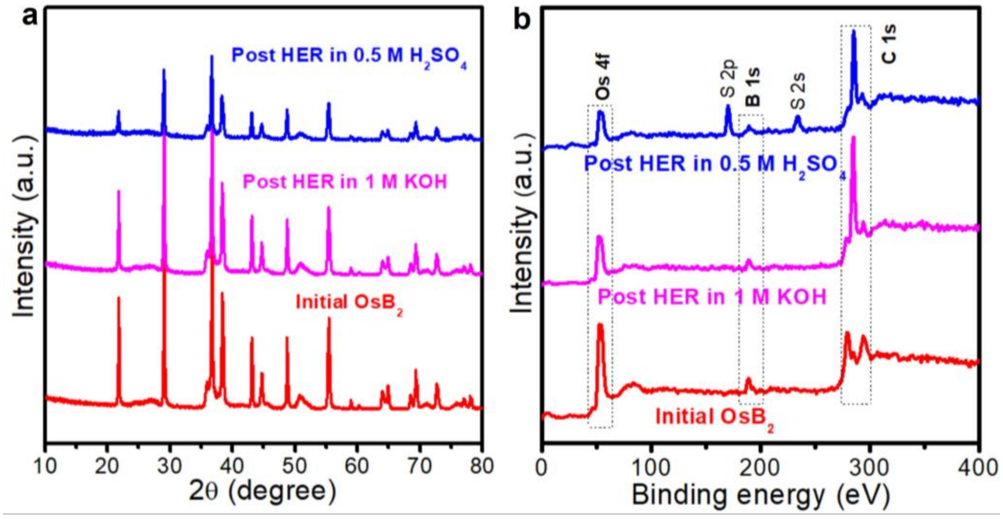

Fig. S27 (**a**) XRD pattern and (**b**) XPS survey of OsB_2 before and after HER electrolysis in 0.5 M H_2SO_4 and 1 M KOH

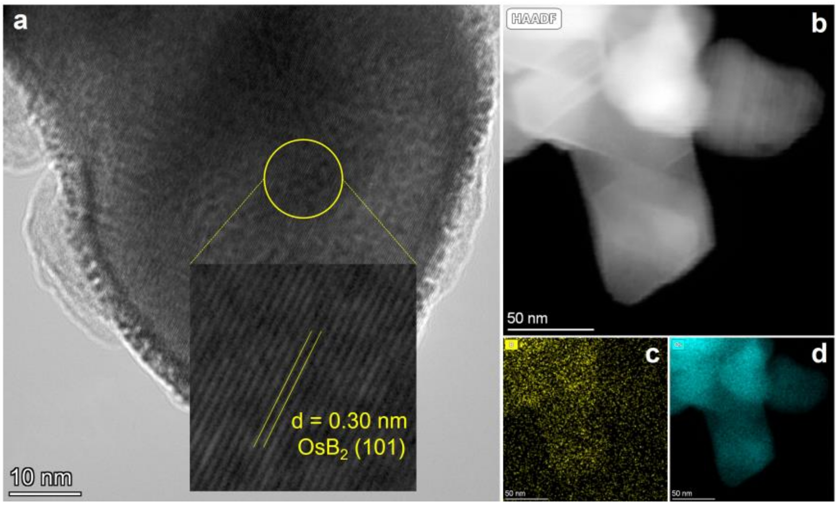

Fig. S28 TEM images of OsB_2 before and after HER electrolysis in 1 M KOH

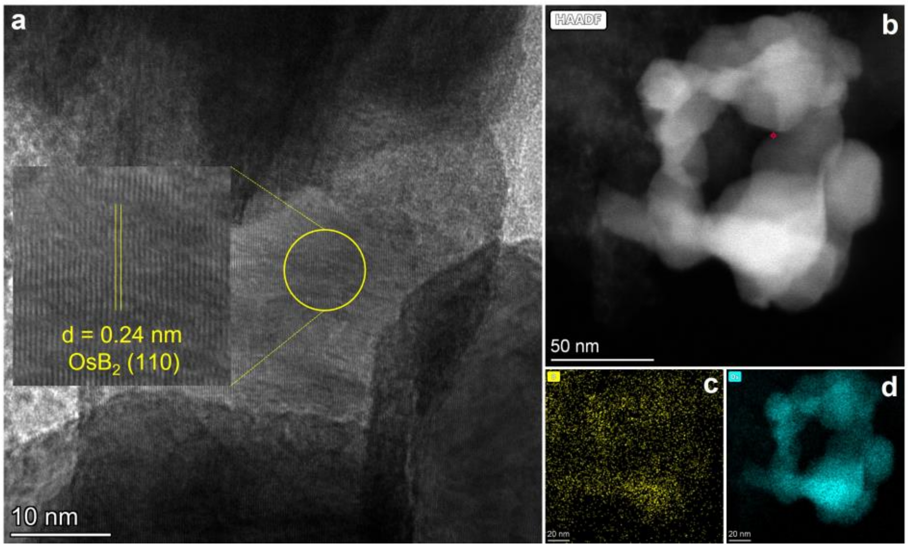

Fig. S29 TEM images of OsB_2 before and after HER electrolysis in 0.5 M H_2SO_4

Table S1 Prices of six platinum group metals[a]

Metal	Symbol	Unit of Measure	U.S.
Platinum	Pt	troy ounce	892
Iridium	Ir	troy ounce	4750
Osmium	Os	troy ounce	378
Palladium	Pd	troy ounce	2110
Rhodium	Rh	troy ounce	14600
Ruthenium	Ru	troy ounce	585

[a] The prices for various platinum group metals are from the BASF corporation website on August 1, 2022. (https://apps.catalysts.basf.com/apps/eibprices/mp/)

Table S2 The content of B in OsB, Os_2B_3 and OsB_2 measured by ICP-OES

Catalyst	Spectrum	Content of B (wt%)
OsB	B 249.677 r	5.6
Os_2B_3	B 249.677 r	8.4
OsB_2	B 249.677 r	11.6

Table S3 The ECSA values of Os, OsB, Os_2B_3 and OsB_2 for HER in 1 M KOH and 0.5 M H_2SO_4

Catalyst	ECSA (cm^2)	ECSA (cm^2)
Os	50.0	36.7
OsB	61.7	41.7
Os_2B_3	73.3	53.3
OsB_2	128.3	75.0

Table S4 Comparison of HER performance of OsB_2 with recently reported noble metal based chemical complex at 10 mA cm^{-2} in alkaline media

Catalyst	Electrolyte	Overpotential (mV)	References
OsB_2	1 M KOH	8	This work
Os_2B_3	1 M KOH	19	This work
OsB	1 M KOH	25	This work
Os	1 M KOH	69	This work
RuS_2	1 M KOH	11	[S8]
RuP_2	1 M KOH	12	[S9]
RhP_2	1 M KOH	18	[S10]
RhB_2	1 M KOH	28	[S11]
IrP_2	1 M KOH	28	[S12]
$RuSe_2$	1 M KOH	30	[S13]
PdP_2	1 M KOH	35	[S14]
PtP_2/Pt	1 M KOH	42	[S15]
Pt/$PtTe_x$	1 M KOH	44	[S16]
PtP_2	1 M KOH	45	[S17]
$IrTe_2$	1 M KOH	54	[S18]
OsP_2@NPC	1 M KOH	70	[S19]
PtSi	1 M KOH	38	[S20]
a-$RuTe_2$ PNRs	1 M KOH	36	[S21]
2D $PtSe_2$	1 M KOH	11	[S22]
h-$RuSe_2$	1 M KOH	34	[S23]
RuP_2@NPC	1 M KOH	54	[S24]
$RuSe_2$@NC	1 M KOH	30	[S25]
$RhSe_2$	1 M KOH	81	[S26]
Li-$IrSe_2$	1 M KOH	72	[S27]

Table S5 EXAFS fitting parameters at the Os L3-edge for various samples

Sample	Shell	N^a	R(Å)^b	σ^2(Å^2)^c	ΔE_0(eV)^d	R factor
Os foil	Os-Os	12.0	2.70	0.0031	8.32	0.0034
OsB	Os-B	6.0	2.19	0.0039	9.23	0.0103
	Os-Os	6.0	2.84	0.0059	5.30	
Os_2B_3	Os-B	7.2	2.20	0.0033	9.84	0.0055
	Os-Os	5.0	2.87	0.0027	5.76	
OsB_2	Os-B	7.2	2.21	0.0070	7.59	0.0148
	Os-Os	3.5	2.89	0.0061	0.38	

[a]CN, coordination number; [b]R, distance between absorber and backscatter atoms; [c]σ^2, Debye-Waller factor to account for both thermal and structural disorders; [d]ΔE_0, inner potential correction; R factor indicates the goodness of the fit. according to the experimental EXAFS fit of Co foil by fixing CN as the known crystallographic value. Fitting range: $3.0 \le k$ (/Å) ≤ 12.5 and $1.0 \le R$ (Å) ≤ 3.0 (Os foil); $3.4 \le k$ (/Å) ≤ 10.4 and $1.2 \le R$ (Å) ≤ 3.1 (OsB) ; $3.0 \le k$ (/Å) ≤ 12.5 and $1.0 \le R$ (Å) ≤ 3.0 (Os_2B_3); $3.0 \le k$ (/Å) ≤ 12.8 and $1.1 \le R$ (Å) ≤ 3.0 (OsB_2).

Supplementary References

[S1] G. Kresse, J. Furthmüller, Efficiency of ab-initio total energy calculations for metals and semiconductors using a plane-wave basis set. Comp. Mater. Sci. **1996**, 6(1): 15-50. https://doi.org/10.1016/0927-0256(96)00008-0

[S2] G. Kresse, J. Furthmüller, Efficient iterative schemes for ab initio total-energy calculations using a plane-wave basis set.. Phys. Rev. B Cond. Matter **1996**, 54(16): 11169. https://doi.org/10.1103/PhysRevB.54.11169

[S3] Matthias Ernzerhof, John P. Perdew, Generalized gradient approximation to the angle- and system-averaged exchange hole. J. Chem. Phys. **1998**, 109(9): 476928. https://doi.org/10.1063/1.476928

[S4] P. E. Blöchl, Projector augmented-wave method. Phys. Rev. B **1994**, 50(24): 17953. https://doi.org/10.1103/PhysRevB.50.17953

[S5] Grimme Stefan, Antony Jens, Ehrlich Stephan, Krieg Helge. A consistent and accurate ab initio parametrization of density functional dispersion correction (DFT-D) for the 94 elements H-Pu. J. Chem. Phys. **2010**, 132(15): 154104. https://doi.org/10.1063/1.3382344

[S6] J. K. Noerskov, T. Bligaard, A. Logadottir et al., Trends in the exchange current for hydrogen evolution. ChemInform **2005**, 36(24): 0524023. https://doi.org/10.1002/chin.200524023

[S7] Er Dequan, Ye Han, Frey Nathan C et al., Prediction of enhanced catalytic activity for hydrogen evolution reaction in janus transition metal dichalcogenides. Nano Lett. **2018**, 18(6): 3943–3949. https://doi.org/10.1021/acs.nanolett.8b01335

[S8] Y. Xu, C. Du, Q. Shen, et al., Well-dispersed pyrite-type RuS_2 nanocrystals anchored on porous nitrogen and sulfur co-doped hollow carbon spheres for enhanced alkaline hydrogen evolution. Chem. Engin. J. **2021**, 417(7): 129318. https://doi.org/10.1016/j.cej.2021.129318

[S9] F. Zhou, R. Sa, X. Zhang, et al., Robust ruthenium diphosphide nanoparticles for pH-universal hydrogen evolution reaction with platinum-like activity. Appl. Catal. B: Environ. **2020**, 274: 119092. https://doi.org/10.1016/j.apcatb.2020.119092

[S10] Wrinkled Rh_2P nanosheets as superior pH-universal electrocatalysts for hydrogen evolution catalysis. Adv. Energy Mater. **2018**, 8(27): 1801891. https://doi.org/10.1002/aenm.201801891

[S11] Q. Li, X. Zou, X. Ai, et al., Revealing activity trends of metal diborides toward pH-universal hydrogen evolution electrocatalysts with Pt-like activity. Adv. Energy Mater. **2019**, 9: 1803369. https://doi.org/10.1002/aenm.201803369

[S12] Z. Pu, J. Zhao, I.S. Amiinu, et al., A universal synthesis strategy for P-rich noble metal diphosphide-based electrocatalysts for the hydrogen evolution reaction. Energy Environ. Sci. **2019**, 12: 952-957. https://doi.org/10.1039/C9EE00197B

[S13] Z. Zhang, C. Jiang, P. Li, et al., Benchmarking phases of ruthenium dichalcogenides for electrocatalysis of hydrogen evolution: theoretical and experimental insights. Small **2021**: e2007333. https://doi.org/10.1002/smll.202007333

[S14] F. Luo, Q. Zhang, X. Yu, et al., Palladium phosphide as a stable and efficient electrocatalyst for overall water splitting. Angew. Chem. Int. Ed. **2018**, 130: 15078-15083. https://doi.org/10.1002/ange.201810102

[S15] Z. Wang, B. Xiao, Z. Lin, et al., $PtSe_2$/Pt heterointerface with reduced coordination for boosted hydrogen evolution reaction. Angew. Chem. Int. Ed. **2021**, 60: 23388-23393. https://doi.org/10.1002/anie.202110335

[S16] J. Chen, M. Qin, S. Ma, et al., Rational construction of Pt/PtTex interface with optimal intermediate adsorption energy for efficient hydrogen evolution reaction. Appl. Catal. B Environ. **2021**, 299: 120640. https://doi.org/10.1016/j.apcatb.2021.120640

[S17] Z. Pu, R. Cheng, J. Zhao, et al., Anion-modulated platinum for high-performance multifunctional electrocatalysis toward HER, HOR, and ORR. iScience **2020**, 23(12): 101793. https://doi.org/10.1016/j.isci.2020.101793

[S18] Yecan Pi, Yong Xu, Leigang Li, et al., Selective surface reconstruction of a defective iridium based catalyst for high efficiency water splitting. Adv. Funct. Mater. **2020**, 30(43): 2004375. https://doi.org/10.1002/adfm.202004375

[S19] L. Fang, Y. Wang, X. Yang, et al., Uniform OsP_2 nanoparticles anchored on N,P-doped carbon: A new electrocatalyst with enhanced activity for hydrogen

generation at all pH values. J. Catal. **2019**, 370: 404-411. https://doi.org/10.1016/j.jcat.2019.01.010

[S20] Z. Pu, T. Liu, G Zhang, General synthesis of transition-metal-based carbon-group intermetallic catalysts for efficient electrocatalytic hydrogen evolution in wide pH range. Adv. Energy Mater. **2022**, 12: 2200293.

[S21] J. Wang, L. Han, B. Huang, et al., Amorphization activated ruthenium-tellurium nanorods for efficient water splitting. Nat. Commun. **2019**, 10(1): 5692. https://doi.org/10.1038/s41467-019-13519-1

[S22] X. Ping, D. Liang, Y. Wu, et al., Activating a two-dimensional PtSe2 basal plane for the hydrogen evolution reaction through the simultaneous generation of atomic vacancies and Pt clusters. Nano Lett. **2021**, 21: 3857–3863. https://doi.org/10.1021/acs.nanolett.1c00380

[S23] W. Luo, Y. Zhao, H. Cong, et al., Hexagonal $RuSe_2$ nanosheets for highly efficient hydrogen evolution electrocatalysis. Angew. Chem. Int. Ed. **2021**, 13: 7089-7093. https://doi.org/10.1002/ange.202016207

[S24] Z. Pu, I.S. Amiinu, Z. Kou, et al., RuP_2 based catalysts with platinum like activity and higher durability for the hydrogen evolution reaction at all pH values. Angew. Chem. Int. Ed. **2017**, 56(38): 11559-11564. https://doi.org/10.1002/anie.201704911

[S25] D. Chen, R. Lu, Y. Yao, et al., Duetting electronic structure modulation of Ru atoms in $RuSe_2$@NC enables more moderate H* adsorption and water dissociation for hydrogen evolution reaction. J. Mater. Chem. A **2022**, 10: 7637-7644. https://doi.org/10.1039/D2TA01032A

[S26] W. Zhong, B. Xiao, Z. Lin et al., $RhSe_2$: A superior 3D electrocatalyst with multiple active facets for hydrogen evolution reaction in both acid and alkaline solutions. Adv. Mater. **2021**: 2007894. https://doi.org/10.1002/adma.202007894

[S27] T. Zheng, C. Shang, Z. He et al., Intercalated iridium diselenide electrocatalysts for efficient pH-universal water splitting. Angew. Chem. Int. Ed. **2019**: 1909369. https://doi.org/10.1002/anie.20
